# A BioLiving Periosteum Evokes Centripetal Regeneration in Challenging Bone Defects

**DOI:** 10.1002/advs.202518401

**Published:** 2026-03-01

**Authors:** Yang Shi, Nian Liu, Jingyi Gu, Yinling Wang, Weiye Wang, Zhiwei Ke, Mingjun Xie, Wenjing Jin, Weicheng Kong, Jue Shi, Hui Pan, Yong He, Zhijian Xie

**Affiliations:** ^1^ Stomatology Hospital, School of Stomatology, Zhejiang University School of Medicine, Zhejiang Provincial Clinical Research Center for Oral Diseases, Key Laboratory of Oral Biomedical Research of Zhejiang Province Cancer Center of Zhejiang University, Zhejiang‐Singapore International Joint Laboratory of Oral Bioengineering Hangzhou China; ^2^ State Key Laboratory of Fluid Power and Mechatronic Systems & Liangzhu Laboratory School of Mechanical Engineering Zhejiang University Hangzhou China; ^3^ Key Laboratory of 3D Printing Process and Equipment of Zhejiang Province College of Mechanical Engineering Zhejiang University Hangzhou China; ^4^ The Second Affiliated Hospital of Zhejiang University Zhejiang University Hangzhou China

**Keywords:** BioLiving periosteum, centripetal bone regeneration, challenging bone defect

## Abstract

Large bone defects often exhibit compromised bone healing in the central region. Natural periosteum serves as a bioactive barrier by preventing unwanted fibroblast infiltration and providing regenerative cells. However, a significant gap remains in obtaining desirable central bone healing in large bone defects, and the challenge of achieving a balance between the dense barrier structure and bioactive functionality remains unresolved. Herein, a BioLiving periosteum is engineered to recapitulate key features of native periosteum. The middle mini‐tissue is obtained by the liquid substrate culture (LSC) method, and encapsulated between a fiber‐guiding layer and a recruitment layer, both of which feature topological structures. The LSC method endows endothelial cells (ECs) with an elevated glycolytic activity, facilitating their transition to a type‐H phenotype, which could secrete multiple growth factors (chemical signals) to recruit endogenous cells. Subsequently, the recruited cells are further guided by the radial topological structure (physical signals) to migrate toward the central area. The combination of remote chemical cues and contact physical cues functions in a signaling‐relay manner, effectively promoting bone formation in the central area of the defect. Thus, the BioLiving periosteum triggers centripetal bone regeneration through a physicochemical signaling‐relay mode, representing a promising therapeutic strategy for severe bone defects.

## Graphical abstract

Large bone defects often suffer from insufficient central healing. This study engineers a BioLiving periosteum that mimics the native structure. It sequentially delivers remote chemical cues and contact physical cues in a signaling‐relay manner, effectively recruiting and guiding endogenous cells toward the defect center. This strategy achieves centripetal bone regeneration, offering a promising therapeutic approach for severe bone defects.

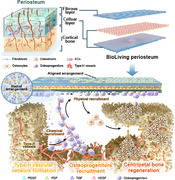

## 1 Introduction

The periosteum is a highly vascularized connective tissue that covers almost the entire bone surface. It acts as the “umbilical cord” of bone tissue and consists of an outer fibrous layer and an inner cambium layer [1]. The dense outer layer involves abundant capillaries and well‐organized aligned collagenous fibers, providing structural support and over 70% blood supply for the underlying bone tissue [2]. The inner layer harbors multiple cell types and various bioactive factors, and provides a critical source of power for bone repair [3]. Growing evidence proved that the periosteum served as a vital conduit for nutrients, biomolecules, and cells to reach injury sites and initiate the healing process [4, 5], and a majority of progenitor cells are originated from the periosteum, especially when endochondral ossification model is involved [6]. Moreover, functional blood vessels, such as type‐H (CD31^hi^ EMCN^hi^) vessels are another pivotal component in the periosteum that couple angiogenesis and osteogenesis during bone regeneration [7, 8, 9], and the damage of periosteum will inevitably lead to up to 10‐fold reduction in vascularization and the delay of bone healing [10, 11]. Therefore, given its decisive role in bone repair, it is crucial to re‐establish multiple biofunctions of the native periosteum, especially for large bone defects due to their central insufficient blood supply and cellular sources.

Another critical role of the native periosteum is to act as a barrier to prevent the invasiveness of epidermal cells and connective tissue cells interfering with osteoblasts, and to selectively promote osteogenesis and angiogenesis [12]. To this end, various natural or synthetic materials have been developed as substitutes for periosteum [13, 14, 15, 16], but these artificial periosteum often involve a compact structure to prevent unwanted tissue ingrowth [17], which inevitably limits nutrient infiltration and cell migration, leading to compromised vascularization and bone formation. Additionally, these synthetic materials typically exhibit poor bioactivity, necessitating the incorporation of exogenous biological factors to accelerate healing [18, 19]. However, rapid or sustained drug release often fails to precisely coordinate with the dynamic changes during bone repair, due to the varying pH and oxidative environments [20, 21, 22]. Also, the limited incorporation of biomolecules targeting specific stages is insufficient to meet the demands of the complex series of events in bone regeneration, including immune modulation, angiogenesis, progenitor recruitment, proliferation, and differentiation [23, 24]. Therefore, developing a living periosteum that can function as a physically protective barrier while also possessing a biologically adaptive mode to meet the dynamic and comprehensive needs of bone regeneration remains a significant challenge.

**SCHEME 1:**
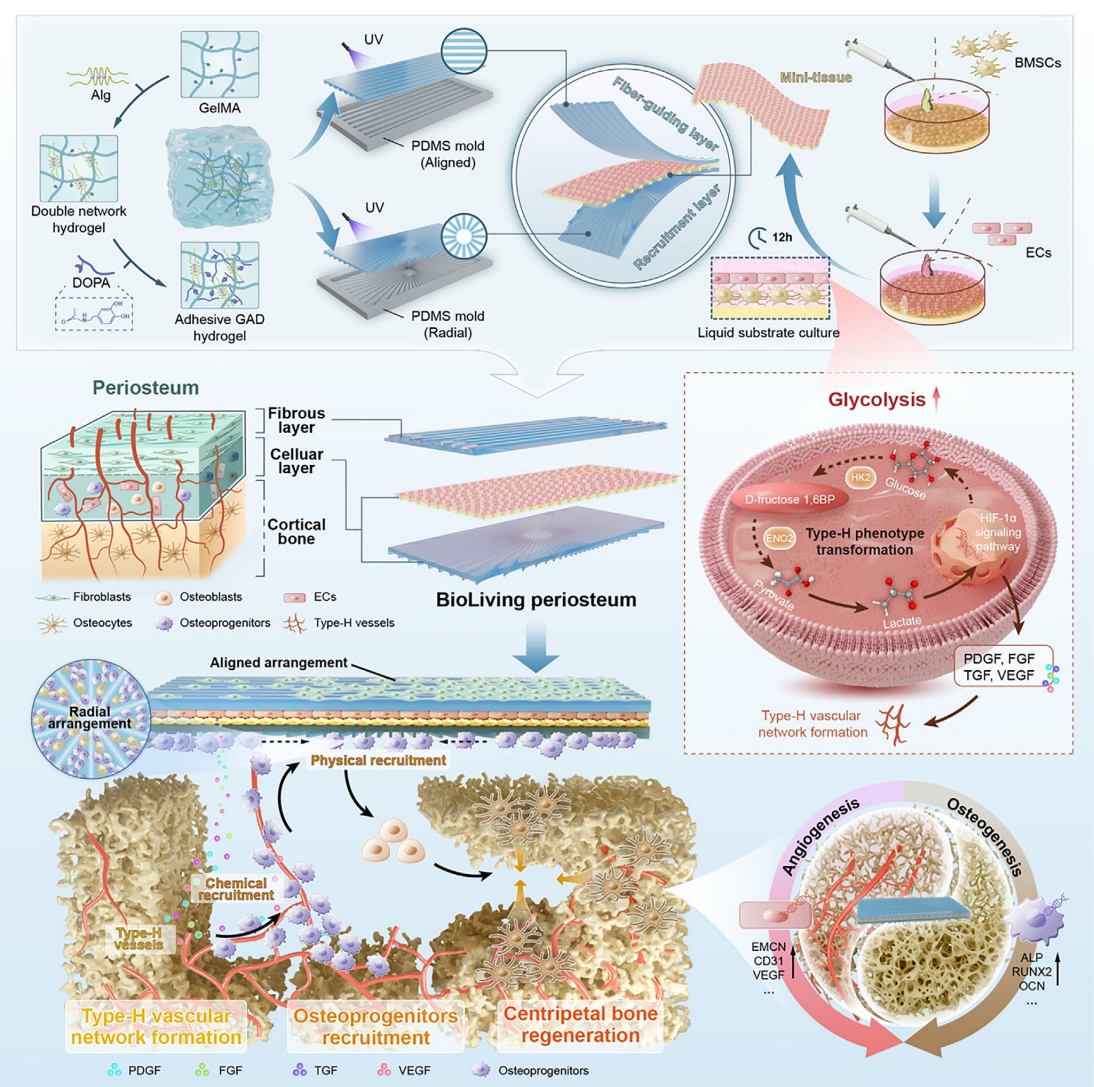
Overview of the BioLiving Periosteum Triggers Centripetal Bone Regeneration. The GAD double network hydrogel system, composed of GelMA, sodium alginate, and DOPA, was patterned using a PDMS mold to create topological features. The upper fiber‐guiding layer with an aligned pattern was specifically designed to guide the arrangement of fibroblasts, while the lower recruitment layer with a radial pattern served as a biophysical signal to recruit osteoprogenitors. A cell‐sheet‐like mini‐tissue was fabricated using the liquid substrate culture (LSC) method, which enhanced the glycolytic activity of endothelial cells (ECs) and promoted their transformation into the type‐H phenotype. In vivo, the type‐H endothelium facilitated the formation of type‐H vascular network, which in turn recruited endogenous osteoprogenitors into the central defect by secreting multiple growth factors (chemical signals). Once recruited, the biological behavior of these cells was further guided by the radial topological structure (physical signals). These physicochemical signals functioned in a signaling‐relay recruitment mode, ultimately achieving centripetal bone regeneration.

Herein, to replicate the natural biological characteristics of the native periosteum (e.g., cell types, bioactive molecules, and topological structures), a BioLiving periosteum, capable of providing various natural biomolecules and functioning as a biological barrier, was developed to recreate a supportive microenvironment for high‐performance bone healing (Scheme 1). These functional features were primarily achieved by a cell‐sheet‐like mini‐tissue, which was generated using the liquid substrate culture (LSC) method, as described in our previous work [25]. This innovative approach enabled anchorage‐dependent adherent cells to spontaneously assemble and form tight intercellular junctions, thereby creating a robust natural barrier. To further replicate the cambium layer of the native periosteum, the mini‐tissue was designed with a double‐layered structure, comprising an endothelial cell (EC) sheet and a bone marrow stromal cell (BMSC) sheet. To provide structural support for this barrier mini‐tissue, a hydrogel system featuring an intricately designed topography was utilized. The upper fiber‐guiding layer was designed with parallel grooves to facilitate the natural alignment of longitudinally oriented fibers, while the inner recruitment layer facing the bone defect featured a radial pattern to attract host progenitor cells into the center of the defect. The resultant BioLiving periosteum could act as a barrier tissue and effectively prevent the infiltration of fibroblasts by the tight cell–cell junction. More importantly, compared with 2D culture, ECs cultured with the LSC method obtained a higher glycolytic activity, this enhanced energy metabolism enabled ECs to adopt a specialized type‐H phenotype with abundant osteogenic and angiogenic biomolecule secretion. As a result, functional type‐H blood vessels were successfully induced in vivo, thereby attracting OSX^+^ osteoprogenitors to the defect site. Subsequently, the recruited cells were guided by the radial pattern of the hydrogel to migrate toward the central region of the defect. These physicochemical cues collectively functioned as remote and contact guidance, promoting functional neovascularization and centripetal bone formation in challenging vertical bone augmentation. Thus, the present study introduced a practical strategy to effectively stimulate the entire intrinsic healing process. The BioLiving periosteum, with its signaling‐relay osteogenic mode, demonstrated significant potential for challenging bone defects.

## 2 Results and Discussion

### 2.1 Fabrication and Characterization of the Supporting Adhesive Dual‐Network Hydrogel

**FIGURE 1:**
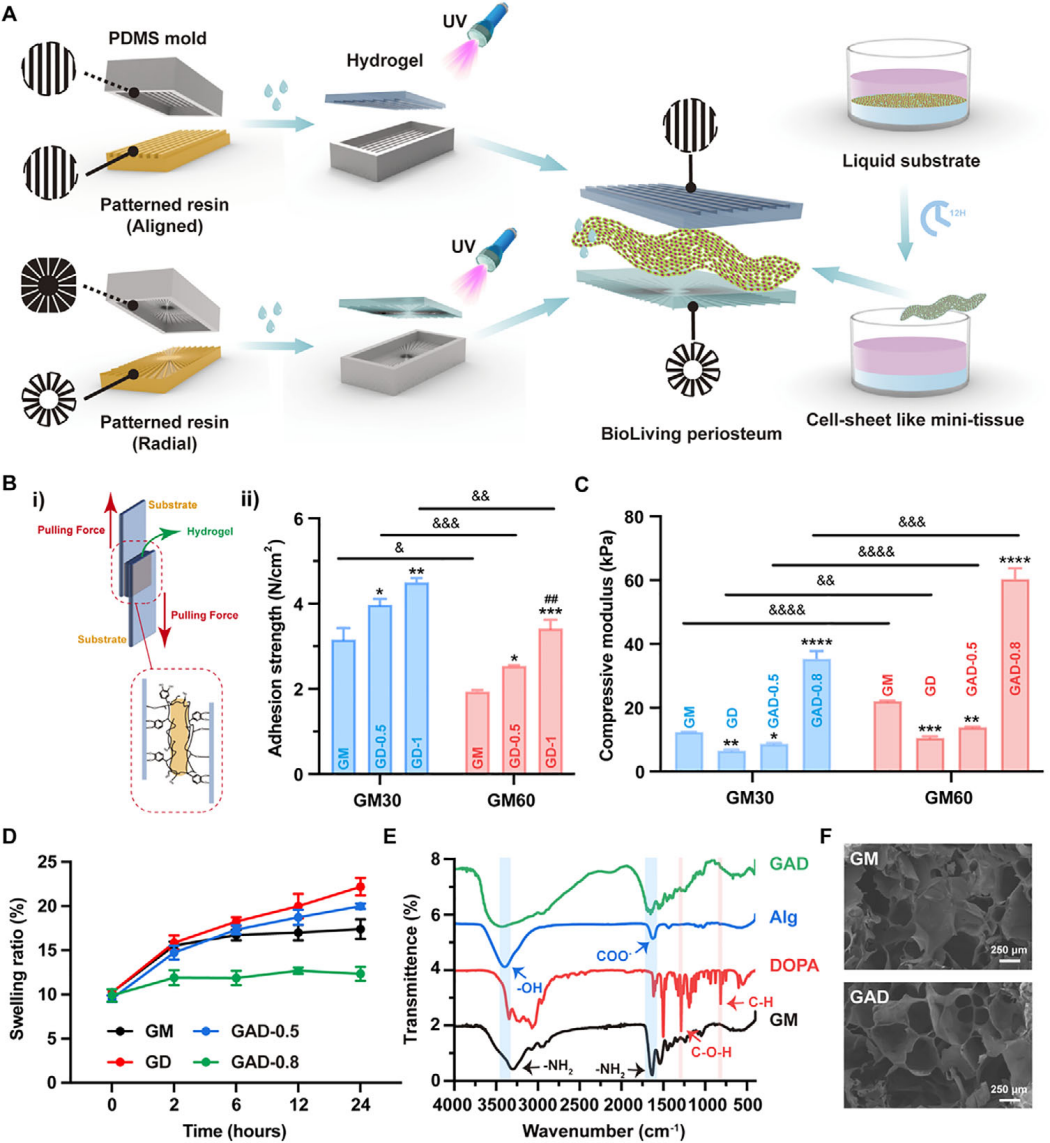
Fabrication and Characterization of the Supporting Hydrogel. (A) Schematic depiction of the fabrication process of the BioLiving periosteum. (Bi) Schematic diagram showing the testing protocol of the shear adhesion strength, and (Bii) quantification of the shear adhesion strength of GelMA 30 (GM30) and GelMA 60 (GM60) loaded with different content (0%, 0.5%, and 1%) of l‐3,4‐dihydroxyphenylalanine (DOPA), respectively (*n* = 3). (C) Quantification of the compressive strength of GM30 and GM60 loaded with or without 1% DOPA and different content (0.5% and 0.8%) of sodium alginate (*n* = 3). (D) The swelling ratio of GM60 loaded with or without 1% DOPA and different content (0.5% and 0.8%) of sodium alginate (*n* = 3). (E) Fourier transform infrared (FTIR) spectroscopy of GelMA (GM), DOPA, sodium alginate (Alg), and GelMA incorporated with sodium alginate and DOPA (GAD). (F) Representative scanning electron microscope (SEM) images of GM and GAD. All statistical data are represented as mean ± SEM. ^*^ *p* < 0.05, ^**^ *p* < 0.01, ^***^ *p* < 0.001, and ^****^ *p* < 0.0001 compared with the GM group. *^##^p* < 0.01 compared with the GD‐0.5 group. Statistical analyses were performed using one‐way ANOVA with Tukey's post hoc test. ^&^ *p* < 0.05, ^&&^ *p* < 0.01, ^&&&^ *p* < 0.001, and ^&&&&^ *p* < 0.001 compared between the GM30 and GM60 groups. Statistical analyses were performed using unpaired two‐tailed Student's *t*‐test.

During the dynamic process of bone healing, the characteristic structure of the periosteum naturally undergoes significant transformations. Upon injury, the periosteum thickens, and the cambium layer develops a distinct beam‐like structure resulting from the proliferation of bone progenitor cells [26]. This intricate topographic surface plays a crucial mechanobiological role in orchestrating cell behavior through contact guidance, ranging from morphological adaptations to functional differentiation [16, 22, 23, 24, [27, 28]. Herein, an extracellular matrix (ECM) mimetic gelatin methacryloyl (GelMA) hydrogel was selected as the foundational backbone [29]. Specifically, inspired by the outer fibrous layer of the native periosteum with longitudinally oriented cells and ECM fibers, a parallelly aligned pattern was designed for the outer fiber‐guiding layer of the BioLiving periosteum (Figure S1A). For the inner recruitment layer, radially oriented microgrooves were designed (Figure S1B), which have been reported to attract the surrounding endogenous cells, guide tissue ingrowth, and facilitate the organized deposition of ECMs [30, 31, 32, 33]. After fabricating the patterned resin templates by the digital light process (DLP) 3D printing method, structural polydimethylsiloxane (PDMS) stencils with either aligned or radial architectures were developed. Subsequently, a hydrogel precursor was dip‐coated onto the PDMS stencils and photo‐crosslinked to achieve the desired structure (Figure 1A). This intricately designed topography may regulate cell alignment and create a biomimetic niche to induce bone regeneration [34].

Generally, an ideal artificial periosteum should possess both adhesion properties and robust operability to eliminate fixation procedures and prevent unwanted tissue integration [35]. To this end, dopamine (DOPA) was employed to enhance the adhesive properties of the cross‐linkable hydrogel, as reported in a previous study [36]. The shear adhesion strength of two GelMA variants, featuring degrees of substitution (DS) 30% and 60%, was assessed at a range of DOPA concentrations, as illustrated in Figure 1Bi). As demonstrated in Figure 1Bii, the incorporation of DOPA increased the adhesive strength in both low DS GelMA (GM30) and high DS GelMA (GM60). Compared with GM60, GM30 exhibited a superior adhesion performance (4.5 N/cm^2^) at a higher DOPA concentration (1 wt%) (Figure S2), probably attributed to the higher abundance of amine and hydroxyl groups remaining in the lower‐substitution GM30. These residual functional groups facilitated hydrogen bonding and π−π interactions with hydroxyl groups in DOPA. However, the mechanical strength of DOPA‐modified GelMA (GD) was reduced as revealed in Figure 1C. This is presumably due to the competitive consumption of free radicals by the catechol structure of DOPA [37], or the occupation of crosslinking sites in the hydrogel by the generated hydrogen bonding and π−π interactions [38], thereby compromising the photo‐crosslinking efficiency. To address the compromised mechanical properties of the GD hydrogel, we introduced sodium alginate to create a dual‐network hydrogel system [39]. This strategy integrated visible light‐crosslinked GelMA with ionically crosslinked alginate (via Ca^2^^+^ coordination), thereby significantly enhancing the compressive modulus [40]. As shown in Figure 1C, the incorporation of 0.8 wt% sodium alginate remarkably enhanced the mechanical properties of both GM30 and GM60. Notably, the higher DS GM60 achieved an acceptable compressive modulus of 60.39 kPa, which was approximately double that of GM30 (Figure S3). Moreover, the swelling ratio decreased significantly with increasing sodium alginate content (Figure 1D). The degradation rate of GelMA was also reduced with the incorporation of DOPA and sodium alginate, particularly under acidic conditions (Figure S4), indicating that the composite hydrogel remained stable in injury environments with lower pH. These results may be attributed to the formation of a denser and more stable dual‐network structure, suggesting that this composite hydrogel system could serve as a reliable supporting hydrogel. Therefore, given its superior adhesive and mechanical properties, GM60 with a higher concentration of DOPA (1 wt%) and sodium alginate (0.8 wt%), designated as GAD, emerged as the most promising candidate for fabricating the BioLiving periosteum, meeting the essential physicochemical requirements necessary to support effective bone reconstruction.

Next, the GAD hydrogel was characterized using Fourier transform infrared (FTIR) spectroscopy, as illustrated in Figure 1E. The presence of aromatic C‐H bending vibrations at 808 cm^−1^ and phenolic C‐O‐H vibrations at 1271 cm^−1^, characteristic of catechol groups, confirmed the incorporation of DOPA and indicated its chemical crosslinking with GelMA [41]. Additionally, the broad absorption band at 3414 cm^−1^ was assigned to the stretching vibrations of ‐OH and ‐NH_2_ groups, while the peak at 1625 cm^−1^ was to ‐COO^−^ (from sodium alginate) or ‐NH_2_ (from GelMA) groups. These findings collectively demonstrated the successful integration of DOPA and sodium alginate into the GelMA hydrogel and highlighted the chemical coordination within this composite system. The scanning electron microscope (SEM) images (Figure 1F) further revealed that the incorporation of DOPA and sodium alginate did not compromise the 3D interconnected porous structure of GAD hydrogel, with a pore size and porosity comparable to those of the GM hydrogel (Figure S5). This favorable architecture meets the biological requirements for osteogenesis [42], and shows potential for further application.

Together, these findings demonstrate that the chemical interactions within the GAD hydrogel system enable the dual‐network hydrogel to exhibit optimal adhesive properties and mechanical behaviors, establishing it as an ideal supporting substrate for the middle mini‐tissue.

### 2.2 Transcriptome Sequencing Reveals Type‐H Phenotypic Transformation of ECs Cultured Using the LSC Method

**FIGURE 2:**
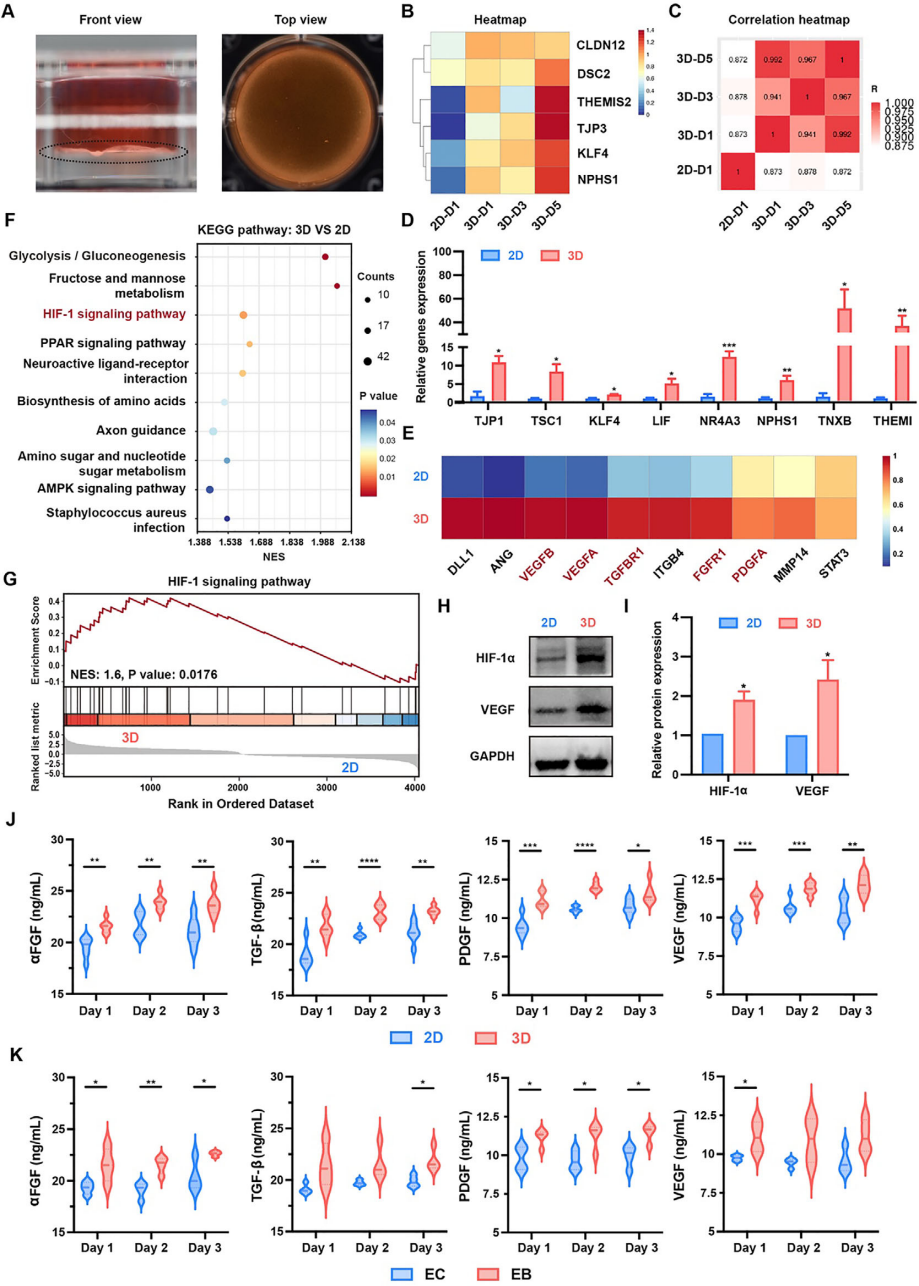
The type‐H phenotypic transformation of endothelial cells (ECs) with the liquid substrate culture (LSC) method. (A) Front and top views of the cell‐sheet‐like mini‐tissue cultured using the LSC method. (B) Heatmap of tight junction‐related genes in the 2D‐cultured group on day 1 (2D‐D1) and 3D‐cultured groups on days 1, 3, and 5 (3D‐D1, 3D‐D3, and 3D‐D5), and (C) the corresponding Pearson's correlation heatmap. (D) The relative expression level of tight junction‐related genes in ECs with 2D and 3D culture (*n* ≥ 3). (E) Heatmap of angiogenesis‐related genes in ECs with 2D and 3D culture on day 1 (red: genes closely associated with type‐H endothelium). (F) Enrichment analysis of Kyoto Encyclopedia of Genes and Genomes (KEGG) pathway for upregulated differentially expressed genes (DEGs). (G) Gene set enrichment analysis (GSEA) analysis of the HIF‐1 signaling pathway. (H) Western blotting analysis for the expressions of HIF‐1𝛼 and vascular endothelial growth factor (VEGF), and (I) the quantitative measurement (*n* = 3). (J) The concentrations of growth factors, including 𝛼FGF, TGF‐β, PDGF, and VEGF, in the supernatant of ECs with 2D and 3D culture on days 1, 3, and 5 (*n* ≥ 3). (K) The concentrations of growth factors, including 𝛼FGF, TGF‐β, PDGF, and VEGF, in the supernatant of cocultured ECs and BMSCs with 2D and 3D culture on days 1, 3, and 5 (*n* ≥ 3). All statistical data are represented as mean ± SEM. Statistical analyses were performed using unpaired two‐tailed Student's *t*‐test. ^*^ *p* < 0.05, ^**^ *p* < 0.01, ^***^ *p* < 0.001, ^****^ *p* < 0.0001 compared with the 2D culture group.

The core cell‐sheet‐like mini‐tissue was fabricated using the LSC method (Figures 1A and 2A), as previously described in our work [25]. This method supported a long‐term culture of mini‐tissue with high cell viability for up to 21 days. Moreover, the resulting 3D mini‐tissue exhibited functional integrity, characterized by selective permeability and self‐healing capabilities, facilitating effective oxygen/nutrient diffusion, thereby serving as a biological barrier. Additionally, we noticed that ECs cultured on the liquid substrate displayed higher expression levels of proangiogenic gene vascular endothelial growth factor (VEGF) compared to those cultured on conventional 2D commercial plates. This observation suggests that the 3D culture environment provided by the LSC method effectively activated the angiogenic potential of ECs.

To further elucidate the phenotypic differences and underlying mechanisms between ECs cultured in 2D and 3D environments, we conducted whole‐transcriptome RNA sequencing (RNA‐seq) analysis. In the gene expression profiles, differentially expressed genes (DEGs) were identified with false discovery rate (FDR) < 0.05 and log_2_FC above cutoff (> 2) sets. As shown in Figure 2B, genes associated with adherens and tight junctions were significantly upregulated in the 3D‐cultured ECs compared to the 2D‐cultured cells, with expression levels increasing over time (Figure 2B). The corresponding correlation heatmap also revealed samples cultured in the 3D environment clustered closely together, demonstrating high correlation among themselves, while the 2D‐cultured sample exhibited low correlation with the 3D samples (Figure 2C). Quantitative real‐time PCR (qRT‐PCR) further confirmed that expression levels of genes associated with tight junctions were significantly upregulated in the 3D‐cultured group compared to the 2D group (Figure 2D). These findings collectively suggested that the LSC method induces tight cell‐to‐cell junctions, which might facilitate cell–cell communication. Moreover, higher expression levels of angiogenesis‐related genes were detected in ECs derived from mini‐tissue compared to those cultured in the 2D environment (Figure 2E and Figure S6). Emerging evidence indicates that type‐H ECs, a specialized subpopulation of endothelium, play a crucial role in modulating osteoblast activity through the production of key biomolecules, which are essential for coupling osteogenesis with angiogenesis during bone repair. Here, we noticed that genes associated with the formation of type‐H vessels, such as VEGF, TGF, FGF, and PDGF, were significantly upregulated in the 3D‐cultured group (Figure 2E). Further analysis using Kyoto Encyclopedia of Genes and Genomes (KEGG) and Gene Set Enrichment Analysis (GSEA) revealed that HIF‐1 signaling pathway, a key pathway involved in the activation of type‐H vessels, was significantly enriched and activated in the 3D group compared to the 2D group (Figure 2F, G). Western blot results further confirmed that HIF‐1𝛼 and VEGF proteins were highly secreted in the 3D group (Figure 2H, I), indicating the HIF‐1𝛼/VEGF signaling pathway was activated in mini‐tissue‐derived ECs. Additionally, the levels of growth factors, including 𝛼FGF, TGF‐β, PDGF, and VEGF, in the supernatant of ECs cultured in both 2D and 3D environments were assessed using enzyme‐linked immunosorbent assay during the first three days of culture. Consistent with the RNA‐seq results, remarkably higher concentrations of these growth factors were detected in the 3D group, and were continued to increase with the prolonged culture time (Figure 2J). Interestingly, when dual ECs and BMSCs were cocultured in a 3D environment (designated as EB), an even greater increase in growth factor concentrations was observed (Figure 2K). This enhanced production was probably attributed to the close crosstalk between those two cell types, including both paracrine signaling and direct cell–cell contact (Figure S7), which further stimulated angiogenesis and osteogenesis at the molecular level.

Collectively, the mini‐tissue fabricated using the LSC method facilitates closer cell–cell interactions, enhances the angiogenic activity of ECs, and increases the secretion of growth factors, particularly inducing their type‐H phenotypic transformation via the HIF‐1𝛼/VEGF signaling pathway. Further, dual cell sheets composed of both endothelial and osteoblast cells may achieve superior angiogenic–osteogenic coupling, thereby synergistically enhancing bone repair. These features are conducive to both the formation of type‐H vessels and the promotion of osteogenesis.

### 2.3 Joint Analysis of Transcriptomics and Metabolomics Reveals Elevated Glycolytic Activity of ECs Supported Type‐H Phenotypic Transformation

**FIGURE 3:**
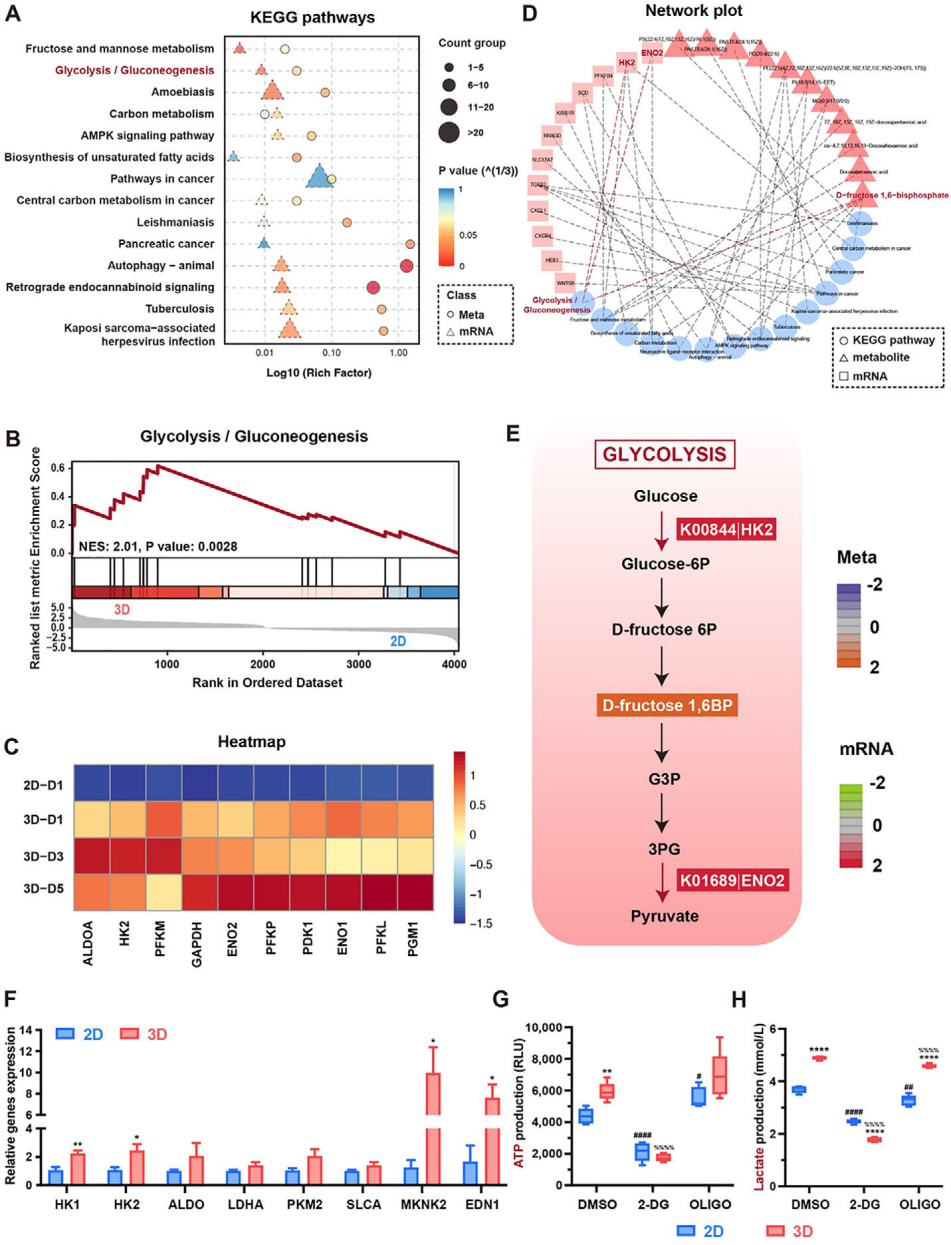
The liquid substrate culture (LSC) method elevated the glycolytic activity of endothelial cells (ECs). (A) Combined pathway enrichment analysis of the 2D and 3D culture groups. (B) Gene set enrichment analysis (GSEA) analysis of the glycolysis/gluconeogenesis pathway. (C) Heatmap of glycolysis‐related genes in the 2D‐cultured group on day 1 (2D‐D1) and 3D‐cultured groups on days 1, 3, and 5 (3D‐D1, 3D‐D3, and 3D‐D5). (D) Network plot showing the correlation between differentially expressed genes (DEGs) and differentially altered metabolites (DAMs) involved in the combined enriched Kyoto Encyclopedia of Genes and Genomes (KEGG) pathways. The glycolysis/gluconeogenesis pathway involved two DEGs (HK2 and ENO2) and one DAM (D‐fructose 1,6‐bisphosphate). (E) Pathway diagram of glycolysis/gluconeogenesis. The red color indicates significantly upregulated expression of genes, and the orange color indicates significantly upregulated metabolites. (F) The relative expression level of glycolysis‐related genes in ECs with 2D and 3D culture (*n* ≥ 3). (G) ATP and (H) lactate levels of ECs with 2D and 3D culture on day 1 (*n* = 5). Cells were treated with 2‐DG and oligomycin (OLIGO), respectively. All statistical data are represented as mean ± SEM. ^*^ *p* < 0.05, ^**^ *p* < 0.01, ^****^ *p* < 0.0001. Statistical analyses were performed using an unpaired two‐tailed Student's *t*‐test. ^#^ *p* < 0.05, ^##^ *p* < 0.01, and ^####^ *p* < 0.0001 compared with the DMSO group with 2D culture, ^%%%%^ *p* < 0.0001 compared with the DMSO group with 3D culture. Statistical analyses were performed using one‐way ANOVA with Dunnett's post hoc test.

Apart from the HIF‐1 signaling pathway, we noticed that glycolysis‐related signaling pathways, such as glycolysis/gluconeogenesis and fructose and mannose metabolism, were significantly enriched (Figure 2F). Similarly, Gene Ontology (GO) and KEGG analysis of genes with upregulated expression over time presented several metabolic terms and pathways, in addition to those related to tight junctions and angiogenesis (Figure S8). These findings indicated that a substantial metabolism shift occurred when ECs were cultured in a 3D microenvironment. To further investigate this metabolic transformation in ECs, metabolomic analysis was conducted. The partial least squares discrimination analysis (PLS‐DA) demonstrated significant overall differences in metabolic profiles between each group, and the permutation test verified the stability and reliability of the PLS‐DA model (R2: 1; Q2: 0.42) (Figure S9A, B). According to the volcano plots, a total of 49 metabolites were significantly increased, while 43 metabolites were decreased in the 3D group compared to the 2D group (Figure S9C). As expected, glycolysis/gluconeogenesis emerged as one of the combined enriched KEGG pathways (Figure 3A). GSEA further revealed that ECs cultured above the liquid substrate exhibited enhanced glycolysis activity compared to those on the conventional plates (Figure 3B), this was also evidenced by the upregulated expressions of glycolysis‐related genes over time (Figure 3C). To elucidate the interplay between transcriptional and metabolic alterations, co‐joint analyses were subsequently conducted to investigate the relationships between DEGs and differentially altered metabolites (DAMs). The network plot identified that two upregulated DEGs‐hexokinase 2 (HK2) and enolase 2 (ENO2)‐as well as one upregulated DAM (D‐fructose 1,6‐bisphosphate, F16BP)‐were implicated in the glycolysis/gluconeogenesis pathway (Figure 3D). These DEGs and DAM were mapped onto the glycolysis pathway, as depicted in Figure 3E. The illustration revealed that when ECs were cultured in a 3D environment, the key glycolytic enzyme HK2 was initially upregulated. This was followed by an enhanced production of F16BP, a core intermediate in glycolysis [43]. Subsequently, the level of downstream glycolytic enzyme ENO2 was elevated. Collectively, these changes drove the glycolysis process.

To further validate the expression levels of key glycolytic genes, such as HK family, aldolase (ALDO), lactate dehydrogenase A (LDHA), pyruvate kinase M2 (PKM2), RT‐qPCR was performed to assess metabolic changes. The results showed all these glycolysis‐associated genes were upregulated in ECs cultured in a 3D environment on day 3, consistent with the findings from RNA‐seq analysis (Figure 3F). These observations implied that the transition from a 2D to a 3D culture environment induced a higher glycolytic rate in ECs. Subsequently, we measured ATP concentration and lactate production of ECs to evaluate their bioenergetic changes. ECs cultured in 3D groups generated significantly more ATP compared to those cultured on traditional plates. Treatment with 2‐Deoxy‐d‐Glucose (2‐DG) markedly reduced ATP levels, whereas oligomycin had minimal impact (Figure 3G), suggesting the primary energy source for ECs was anaerobic glycolysis in either 2D or 3D environment, which was in line with the existing reports [44]. Correspondingly, 3D culture mode led to significantly higher lactate production, supporting the increased ATP generation. Particularly, in the 3D group, the lactate level was reduced by more than half with 2‐DG treatment compared to the control group, while oligomycin treatment had little effect on lactate production (Figure 3H). Further, when treated with 2‐DG, ECs cultured in 3D environment represented downregulated HIF‐1𝛼 and VEGF expression (Figure S10), accompanied by the reduced secretion of representative type‐H growth factors (Figure S11). These results further confirmed that ECs within the mini‐tissue exhibited an elevated glycolytic phenotype compared with the 2D culture environment; this metabolic change facilitates the transformation of ECs into oxygen‐rich type‐H ECs, which is associated with increased bioenergetic and biosynthetic demands to support efficient oxygen transport.

Therefore, the 3D culture environment enhances the glycolytic state of ECs by upregulating a series of key glycolytic enzymes and metabolites. This elevated glycolytic activity increases ATP production, thereby meeting the heightened bioenergetic demands associated with the type‐H endothelial phenotype and supporting the transformation of ECs into type‐H ECs.

### 2.4 The Mini‐Tissue Serves as an Effective Biological Barrier Against Fibroblast Infiltration

**FIGURE 4:**
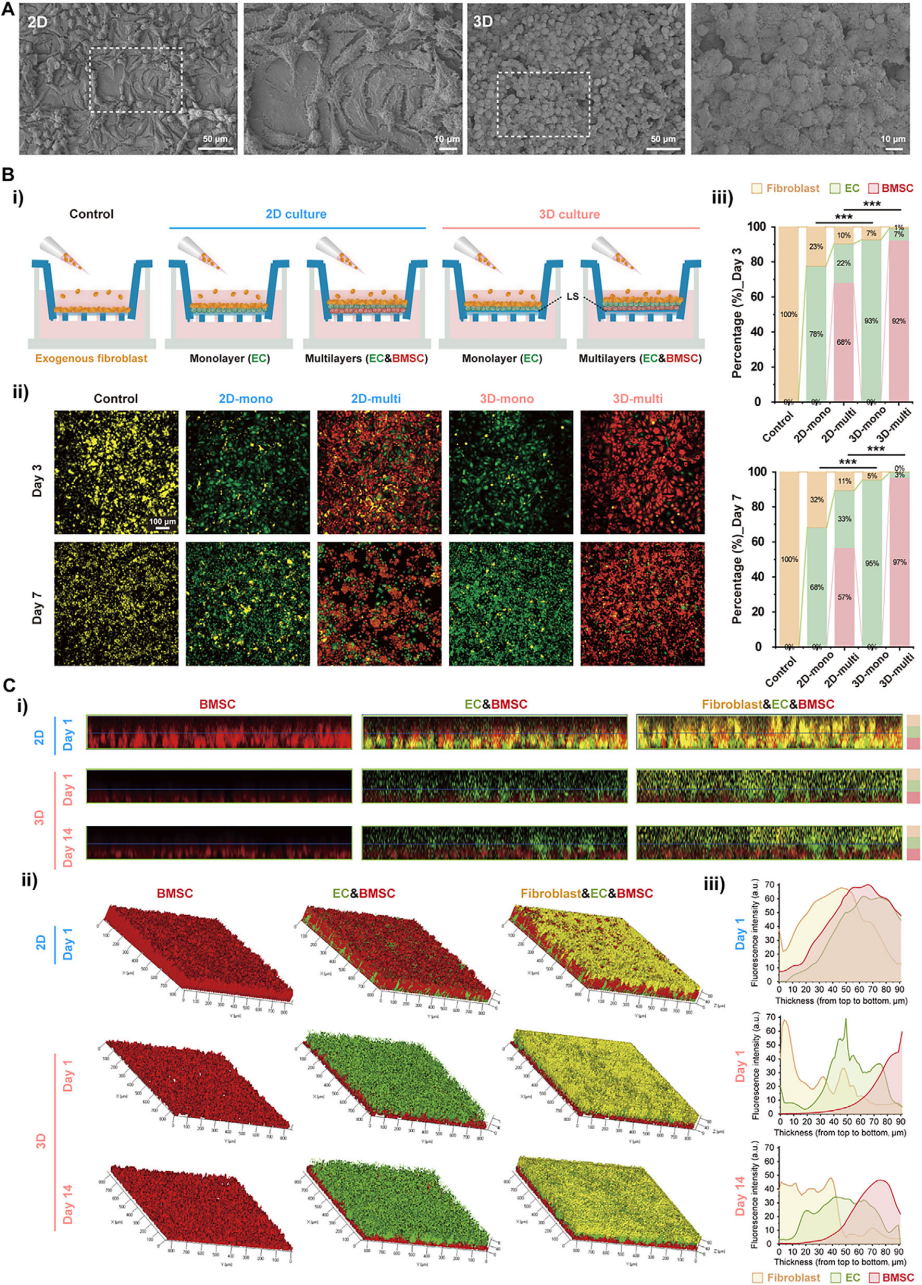
The barrier function evaluation of the cell‐sheet‐like mini‐tissue. (A) Scanning electron microscope (SEM) images of endothelial cells (ECs) cultured with the conventional plate (2D) and the liquid substrate culture (LSC) method (3D), respectively, at different magnifications. (Bi) Schematic diagram of fibroblast infiltration test using a Transwell system. ECs with (multi) or without (mono) bone marrow stromal cells (BMSCs) were sequentially seeded onto the membrane directly or indirectly (with fluorinated oils as a bedding layer), and exogenous fibroblasts were then seeded above these cell sheets, with the exogenous fibroblasts directly seeded onto the membrane as the control. (Bii) Fluorescence microscopy images of exogenous fibroblasts migration through cell sheets (yellow: fibroblasts; green: ECs; red: BMSCs), and (Biii) the corresponding quantitative measurement on days 3 and 7. (Ci) 2D and (Cii) 3D confocal images of ECs in 2D and 3D groups on days 1 and 14, and (Ciii) the corresponding quantitative measurement. All statistical data are represented as mean ± SEM. ^***^ *p* < 0.001. Statistical analyses were performed using an unpaired two‐tailed Student's *t*‐test.

During the bone healing process, a protective barrier is essential for preventing fibroblast invasion and establishing a stable, regenerative osteogenic environment. As presented in Figure 4A, cells in a traditional 2D culture environment exhibited distinct intercellular gaps. In contrast, cells cultured above the liquid substrate formed a tightly packed three‐dimensional structure with direct cell‐to‐cell contact. This structural arrangement is conducive to serving as a barrier against fibroblast infiltration. Further, when seeded above the EC sheet, BMSCs and fibroblasts also exhibited similar morphologies (Figure S12 ***)***. The barrier function of mini‐tissue was initially evaluated using a Transwell system, with fluorinated oil placed in the upper chamber. Both single EC sheet (monolayer) and dual EC and BMSC sheets (multilayers) were tested, as illustrated in Figure 4Bi. Compared to cells directly seeded onto the membrane, cell sheets formed above liquid substrate significantly reduced the migration of exogenous fibroblasts from the upper chamber, as shown in Figure 4Bii, with minimal yellow‐labeled fibroblasts observed. Moreover, cell sheets with multilayers exhibited superior barrier function, with virtually no fibroblast infiltration across the membrane even on day 7. However, in a 2D culture environment, over one‐third of fibroblasts were able to invade the EC layer after 7 days. Although a dual‐layer structure composed of ECs and BMSCs could inhibit fibroblast migration, its effectiveness was limited (Figure 4Biii, and Figure S13). To directly visualize the infiltration behavior of fibroblasts across cell layers, mono‐ or multilayered cell constructs were seeded either in a traditional plate or on liquid substrate. Confocal images revealed that when cells were sequentially seeded above the liquid substrate, each layer maintained a distinct distribution even after 14 days (Figure 4C, and Figure S14). In contrast, in the 2D environment, cells migrated and intermingled with other layers by day 1; this is probably attributed to the distinct intercellular gaps as detected on SEM images in Figure 4A. These results confirmed that the mini‐tissue fabricated using the LSC method exhibited a dense morphology capable of resisting fibroblast migration. In addition, multilayers composed of ECs and BMSCs exhibited superior barrier function compared with a monolayer EC sheet.

Therefore, the tight cell‐to‐cell junctions are ideally preserved by the LSC method without involving any disruptive manipulation; this tight contact functions as an effective biological barrier against fibroblast infiltration.

### 2.5 Physicochemical Cues Modulate Cellular Behavior in a Signaling Relay Manner

**FIGURE 5:**
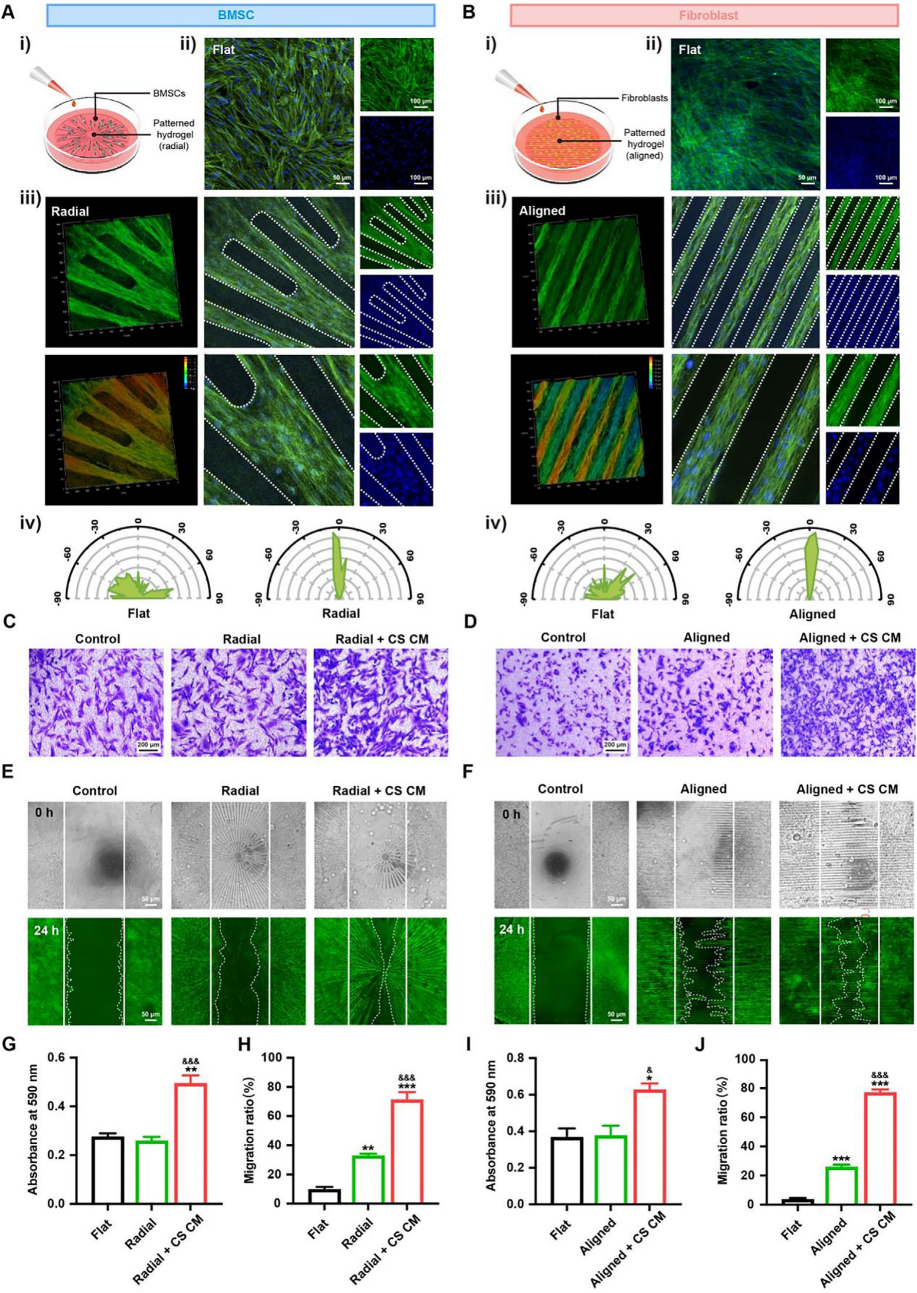
Migration and arrangement behaviors of bone marrow stromal cells (BMSCs) and fibroblasts. (Ai) Schematic diagram of BMSCs seeded onto the hydrogel disks. Confocal images of BMSCs seeded onto the (Aii) Flat and (Aiii) Radial patterned hydrogels. The dotted white line indicates the topological structure of the hydrogel. (Aiv) Quantitative evaluation of the directionality of BMSCs above both hydrogels. (Bi) Schematic diagram of fibroblasts seeded onto the hydrogel disks. Confocal images of fibroblasts seeded onto the (Bii) Flat and (Biii) Aligned patterned hydrogels. The dotted white line indicates the topological structure of the hydrogel. (Biv) Quantitative evaluation of the directionality of fibroblasts above both hydrogels. (C) The migration behavior of BMSCs seeded on the flat or radial pattern hydrogel with or without cell sheet conditioned medium (CS CM) treatment using the Transwell system at 0 and 24 h, and (G) the quantitative measurement (*n* = 3). (D) The migration behavior of fibroblasts seeded on the flat or aligned pattern hydrogel with or without CS CM treatment using the Transwell system at 0 and 24 h, and (I) the quantitative measurement (*n* = 3). (E) Representative images of the migration behavior of BMSCs seeded directly on the hydrogel in different groups with or without CS CM treatment, and (H) the quantitative measurement (*n* = 5). (F) Representative images of the migration behavior of fibroblasts seeded directly on the hydrogel in different groups with or without CS CM treatment, and (J) quantitative measurement (*n* = 5). All statistical data are represented as mean ± SEM. Statistical analyses were performed using one‐way ANOVA with Tukey's post hoc test. ^*^ *p* < 0.05, ^**^ *p* < 0.01, and ^***^ *p* < 0.001 compared with the Flat group. ^&^ *p* < 0.05 and ^&&&^ *p* < 0.001 compared with the radial/aligned group.

To explore the cellular behavior guidance of the designed hydrogel, BMSCs and fibroblasts were seeded above respectively to mimic in vivo conditions. Compared to the hydrogel with no pattern structure (Flat), BMSCs and fibroblasts exhibited well‐organized orientation in radial and aligned patterns, respectively (Figure 5A, B, and Movies S1 and S2). Subsequently, wound healing experiments were conducted to evaluate the migration behavior of both cells. The Transwell assay was initially used to assess the three‐dimensional migration ability. Since cells were not in direct contact with the patterned hydrogels, biophysical cues of hydrogels did not influence their three‐dimensional migration. Interestingly, when treated with conditioned medium from dual cell sheets, significantly more BMSCs and fibroblasts migrated from the upper to the lower chambers (Figure 5C, D, G, I). This enhanced migration might be attributed to the growth factors secreted by the cell sheets, as demonstrated in Figure 2K. Then, we seeded cells directly onto hydrogel disks to investigate the combined effects of biophysical and biochemical cues. After 3 days of culture, the titanium strip, which had been used to prevent cell adhesion to the central area, was removed. Compared to the flat hydrogel, BMSCs in the radial‐patterned hydrogel disks exhibited an evident migration at 24 h. When cotreated with the conditioned medium from dual cell sheets in the radial + CS group, the gap was almost completely closed (Figure 5E, H). A similar phenomenon was also observed for fibroblasts cultured in the aligned‐patterned hydrogel (Figure 5F, J). These results suggested that native cells in defects might initially respond to biochemical cues from the implanted materials. After migrating to the targeted area, biophysical cues continued to regulate the spatial arrangement of these cells.

Taken together, the biochemical cues (biomolecules secreted from the mini‐tissue) and biophysical cues (topological patterns designed on the hydrogels) function in a signaling relay manner to synchronously modulate the migration and arrangement of BMSCs and fibroblasts.

### 2.6 The BioLiving Periosteum Optimizes the Biological Behavior of BMSCs In Vitro

**FIGURE 6:**
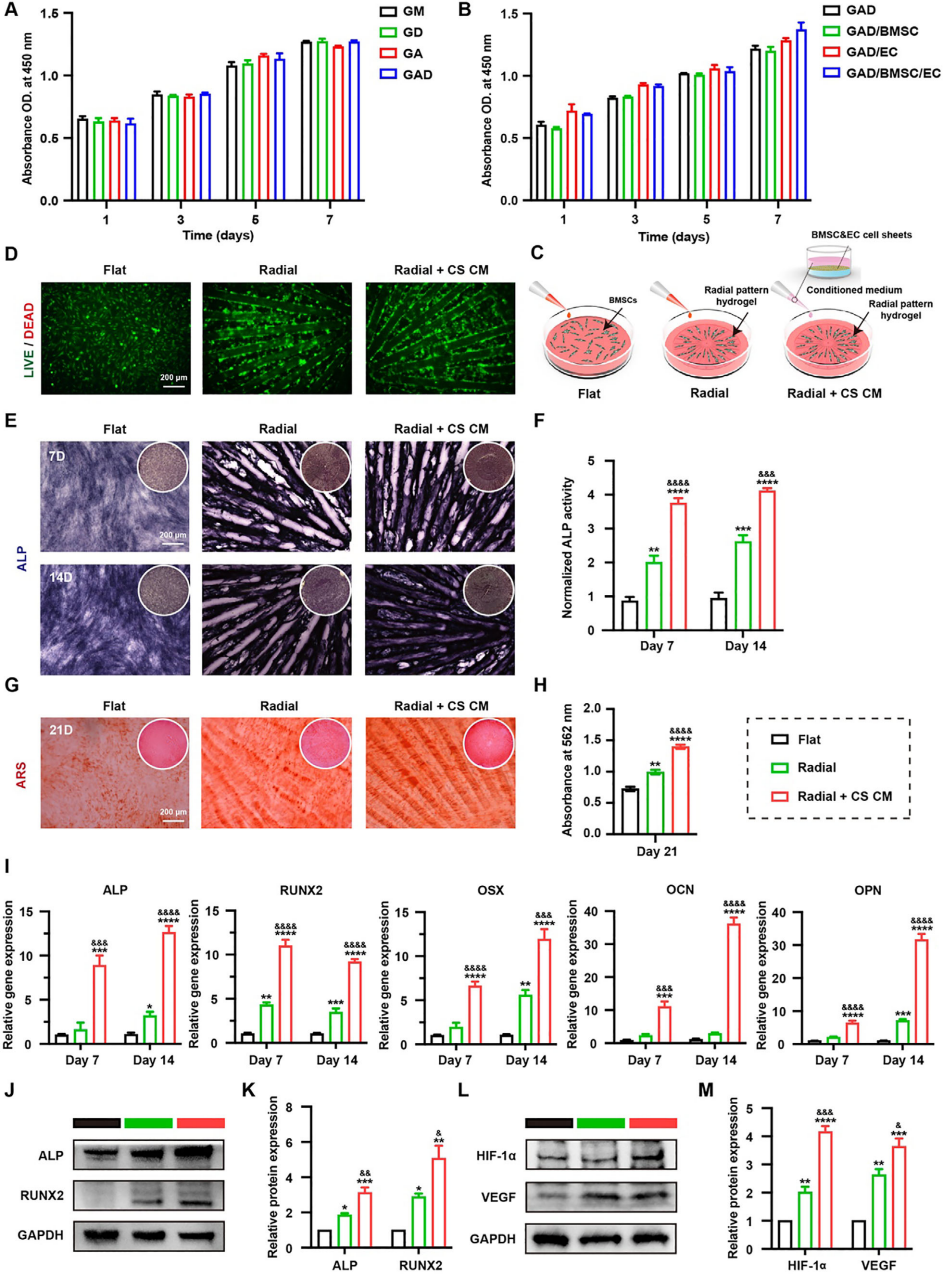
Cytocompatibility and bioactivity evaluation of bone marrow stromal cells (BMSCs). (A) CCK8 analysis of BMSCs seeded on various hydrogels for 1, 3, 5, and 7 days (*n* = 3). GM: GelMA, GD: GelMA with DOPA, GA: GelMA with sodium alginate, GAD: GelMA with sodium alginate and DOPA. (B) CCK8 analysis of BMSCs seeded on GAD hydrogel cocultured with conditioned medium from single‐layered cell sheet (BMSC or EC) or dual‐layered cell sheets (BMSC and EC) for 1, 3, 5, and 7 days (*n* = 3). (C) Schematic diagram of BMSCs seeded above the flat or radial patterned hydrogel with or without conditioned medium from dual cell sheets (CS CM) treatment. (D) Live/dead staining of BMSCs cultured in different groups on day 3 (Green: live cells; red: dead cells). (E) Gross view (inset) and microscopic images of alkaline phosphatase (ALP) staining for BMSCs cultured in different groups at days 7 and 14, and (F) the quantitative measurement (*n* ≥ 3). (G) Gross view (inset) and microscopic images of Alizarin Red S (ARS) staining for BMSCs cultured in different groups at day 21, and (H) the quantitative measurement (*n* = 4). (I) The osteogenic genes expression of BMSCs in different groups at days 7 and 14 (*n* ≥ 3). (J) Western blotting analysis for the expression of ALP and RUNX2, and (K) the quantitative measurement (*n* = 3). (L) Western blotting analysis for the expression of HIF‐1𝛼 and VEGF, and (M) the quantitative measurement (*n* = 3). All statistical data are represented as mean ± SEM. Statistical analyses were performed using one‐way ANOVA with Tukey's post hoc test. ^*^ *p* < 0.05, ^**^ *p* < 0.01, ^***^ *p* < 0.001, and ^****^ *p* < 0.0001 compared with the Flat group. ^&^ *p* < 0.05, ^&&^ *p* < 0.01, ^&&&^ *p* < 0.001, and ^&&&&^ *p* < 0.0001 compared with the Radial group.

To investigate the biocompatibility of the composite hydrogels, BMSCs were initially seeded onto plain hydrogel disks from different groups. Cell proliferation, assessed using the CCK8 assay, increased with extended culture time (Figure 6A). Similar trends were also observed in fibroblasts and ECs (Figure S15A, B). Subsequently, conditioned medium from BMSC sheet, EC sheet, and dual cell sheets was added to culture BMSCs seeded on GAD hydrogel disks, respectively. The results indicated that all conditioned media supported the survival and proliferation of BMSCs (Figure 6B). These findings demonstrated that both composite hydrogels and conditioned medium possessed excellent biocompatibility. Further investigation into the biological effects of topographic and biochemical cues was conducted, as illustrated in Figure 6C. Similarly, high cell viability was observed in all groups (Figure 6D). The osteogenic activities of BMSCs in different groups were first evaluated using alkaline phosphatase (ALP) staining and Alizarin Red S (ARS) staining. ALP, a critical biomarker of osteogenic differentiation, promotes bone formation by catalyzing the hydrolysis of pyrophosphate. As shown in Figure 6E, when cultured in an osteoinductive medium, all groups exhibited positive ALP staining at both 7 and 14 days. The radial structure of hydrogel not only guided the alignment behavior of BMSCs, but also enhanced their osteogenic potential. Furthermore, when cocultured with conditioned medium from dual cell sheets, ALP activity was the most pronounced at day 7 and persisted until day 14. ARS staining revealed increased calcium deposition in the Radial and Radial + CM groups, with the highest level of mineralization observed in the Radial + CM group, as evidenced by both gross appearance and microscopic analysis (Figure 6G). Quantitative analyses of ALP and ARS staining further verified these findings (Figure 6F, H). To elucidate the molecular mechanisms underlying these effects, qRT‐PCR and western blot analyses were performed. As shown in Figure 6I, both early (including ALP, RUNX2, and OSX) and late osteogenic markers (including OCN and OPN) were significantly upregulated in the Radial and Radial + CM groups compared to the control group, though with varying degrees of increase. Notably, the addition of conditioned medium led to a remarkable increase from day 7 to day 14. Correspondingly, western blot also revealed that ALP and RUNX2 protein levels were markedly elevated in BMSCs cultured on patterned hydrogels, and the conditioned medium further enhanced their osteogenic differentiation. The darkest and thickest bands for ALP and RUNX2 were observed in the Radial + CM group, as shown in Figure 6J, which was further verified by subsequent quantitative analyses (Figure 6K).

Extensive studies have revealed that the coupling of the vascular and skeletal system plays a crucial role in bone regeneration [45]. Osteoblast lineage cells are recognized as one of important sources of VEGF within the regenerative niche [46, 47, 48], which stimulates both osteogenic and angiogenic potential of regenerative cells. During the bone healing process, osteoblast‐derived VEGF also acts on adjacent ECs to mediate mitogenic and angiogenic effects, playing a vital role in maintaining vascular integrity and bone mass [49, 50]. Therefore, we also investigated the protein expressions of HIF‐1α and VEGF to assess the angiogenic potential of BMSCs. The expression of HIF‐1α and its most critical downstream effector VEGF were significantly upregulated by the topographical cues, and further increased by incubation with conditioned medium from dual cell sheets (Figure 6L, M). This finding was consistent with previous studies [51, 52], which demonstrated that the 3D topographical structure could upregulate the HIF‐1α signaling pathway, thereby activating the expression of VEGF in BMSCs.

Hence, the radial architecture significantly stimulates the osteogenic and angiogenic behavior of BMSCs, and this biofunction is further enhanced when cocultured with conditioned medium from dual cell sheets, indicating that the combined BioLiving periosteum could create a constructive microenvironment conducive to coupling osteogenesis and angiogenesis.

### 2.7 The BioLiving Periosteum Potentiates Angiogenesis and Osteogenesis in Calvarial Defect

**FIGURE 7:**
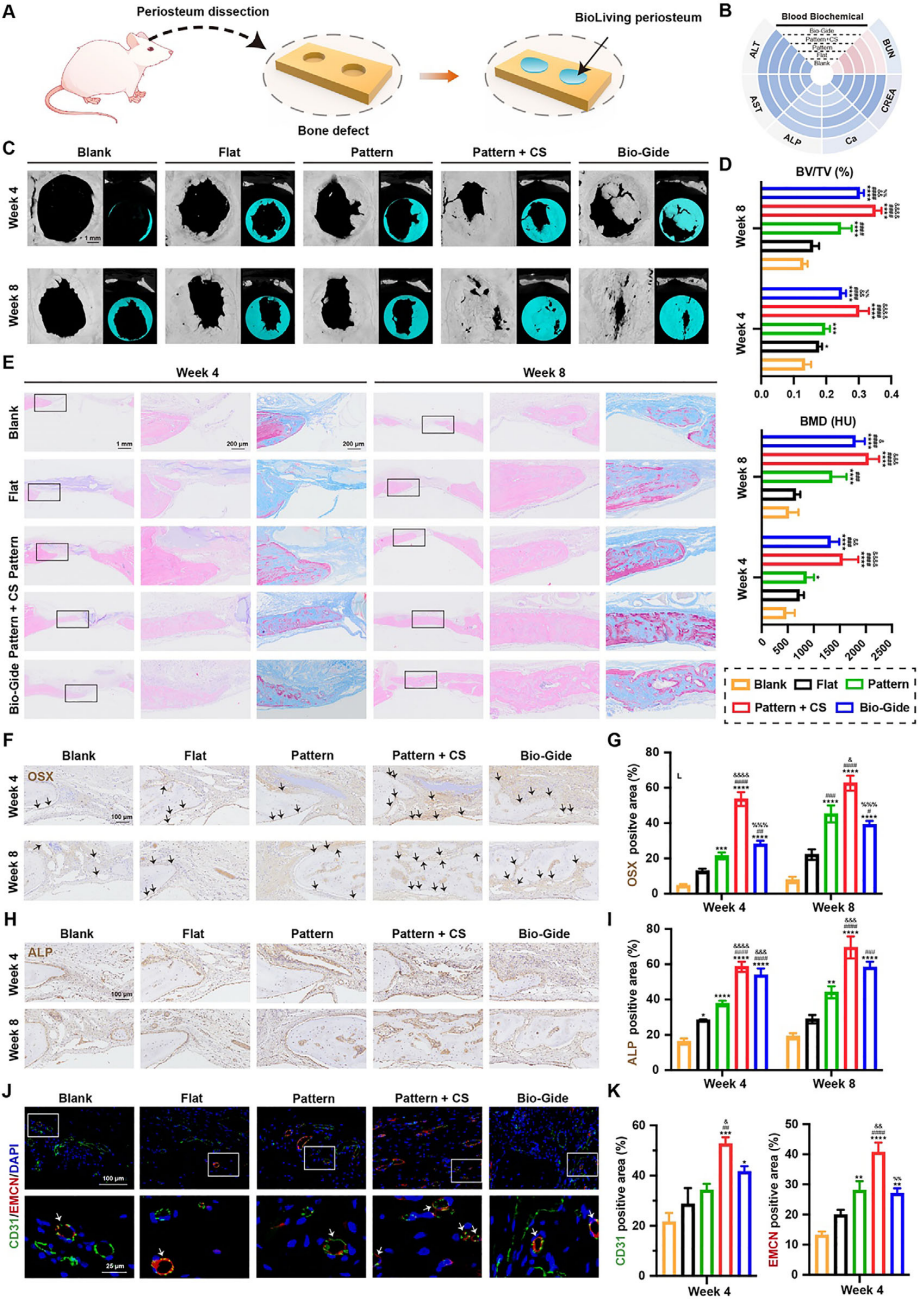
In vivo therapeutic performance of the BioLiving periosteum in calvarial defect. (A) Schematic diagram of the surgery process and periosteum implantation. (B) Serum biochemical analysis of ALT, AST, ALP, BUN, CREA, and Ca (*n* = 6). (C) 2D and 3D micro‐CT images of defects at 4 and 8 weeks after treatment. (D) Quantitative analysis of bone volume/total volume (BV/TV) and bone mineral density (BMD) (*n* ≥ 5). (E) H&E staining and Masson's trichrome staining of calvarial defects after 4 and 8 weeks of treatment. (F) Representative micrographs of IHC staining of OSX (the blank arrows indicated OSX^+^ cells), and (G) the semiquantitative analysis (*n* = 5). (H) Representative micrographs of IHC staining of ALP, and I) the semiquantitative analysis (*n* = 5). (J) Representative images of multi‐immunofluorescence staining for type‐H vessels (CD31^hi^ EMCN^hi^) grown in defect areas (the white arrows indicated CD31^hi^ EMCN^hi^ vessels), and (K) the semiquantitative analysis of CD31 and EMCN (*n* = 5). All statistical data are represented as mean ± SEM. Statistical analyses were performed using one‐way ANOVA with Tukey's post hoc test. ^*^ *p* < 0.05, ^**^ *p* < 0.01, ^***^ *p* < 0.001, and ^****^ *p* < 0.0001 compared with the Blank group. ^#^ *p* < 0.05, ^##^ *p* < 0.01, ^###^ *p* < 0.001, and ^####^ *p* < 0.0001 compared with the Flat group. ^&^ *p* < 0.05, ^&&^ *p* < 0.01, ^&&&^ *p* < 0.001, and ^&&&&^ *p* < 0.0001 compared with the Pattern group. ^%%^ *p* < 0.01, and ^%%%^ *p* < 0.001 compared with the Pattern + CS group.

A standard critical‐sized calvarial defect model was established in rats to evaluate in vivo functions of the BioLiving periosteum. Surgical procedures were illustrated in Figure 7A. After creating bone defects, the BioLiving periosteum (Pattern + CS) was used to cover the defect, compared with the Pattern (periosteum without dual cell sheets) and the Flat group (periosteum without dual cell sheets and topographical structure). Meanwhile, the Bio‐Gide membrane, commonly used in clinical settings, was employed as a positive control, and the Blank group (without any treatment) served as the negative control. Systemic toxicity was first assessed by analyzing blood biochemical indicators, including ALT, AST, ALP, Ca, CREA, and BUN. All indicators were within the normal reference range and showed no significant differences among all groups (Figure 7B).

At 4 and 8 weeks post‐implantation, the animals were sacrificed, and micro‐CT scanning was performed to evaluate mineralized tissue formation (Figure 7C). The Blank group without any treatment exhibited minimal bone regeneration at the defect edge even after 8 weeks. In contrast, bone formation was significantly increased in groups treated with different artificial periosteum. Specifically, periosteum with surface topography demonstrated enhanced new bone formation rates compared to the Flat group. Moreover, the involvement of dual cell sheets within the periosteum further improved osteogenic performance. Notably, by the end of 8 weeks, the Pattern + CS group achieved the most substantial bone formation, with a bony bridge beginning to cover the defect area. In comparison, the Bio‐Gide group showed insufficient bridging, despite partial infiltration of bone tissue into the defect center. Quantitative analysis revealed that the Pattern + CS group exhibited the highest bone volume/total volume (BV/TV) and bone mineral density (BMD), even surpassing the commercial Bio‐Gide group (Figure 7D). This superior performance might be attributed to the osteoinductive function of biomolecules secreted by the dual cell sheets. In contrast, Bio‐Gide, which is primarily composed of collagen, lacks such biochemical signals.

Histological analysis was conducted using H&E and Masson staining to assess the microstructure of the regenerated bone tissues (Figure 7E). At 4 weeks, fibrous tissue was observed in all groups, with minimal new bone formation along the defect margins in the Blank and Flat groups. In contrast, the Pattern + CS and Bio‐Gide groups exhibited a combination of woven and lamellar bone. Notably, the bone regenerated by the BioLiving periosteum was thicker and denser, while the Bio‐Gide group demonstrated a more porous microstructure. A significant amount of residual periosteum was observed in all groups except for the untreated Blank group, with new bone tissue forming beneath the fibrotic tissue and the remaining periosteum. By 8 weeks, as anticipated, the remnant periosteum had degraded to fragments. In the Pattern + CS group, bone defects were almost repaired by the BioLiving periosteum, including the defect center. In contrast, the bony bridge was not fully connected in other groups. Similarly, the newly formed bone guided by the Bio‐Gide membrane was more porous and stained with blue immature collagen fibers. Conversely, the Pattern + CS group displayed a transition from blue to red, indicating the BioLiving periosteum had stimulated a substantial amount of mature and mineralized bone formation within the defect area at this time point.

Immunohistochemistry analyses were carried out to provide additional insights into the osteogenic role of the BioLiving periosteum. OSX is an early indicator of osteogenic differentiation during the bone repair process. In the Pattern group, the number of OSX^+^ osteoprogenitor cells significantly increased in the presence of topological cues. As indicated by the in vitro migration experiment, this might be attributed to the radial structure promoting the colonization of OSX^+^ cells into the defect center. Notably, the introduction of dual cell sheets created a high concentration of various growth factors in the niche, resulting in the greatest number of OSX^+^ cells being attracted and observed around the BioLiving periosteum (Figure 7F, G). When osteoblasts began to form bone, they secreted abundant ECM components, such as ALP. As shown in Figure 7 (H, I), the implantation of periosteum enhanced ALP secretion in the bone matrix compared to the non‐treatment group at both 4 and 8 weeks, and the BioLiving periosteum group exhibited the highest ALP expression.

To further evaluate whether type‐H vessel angiogenesis was induced in vivo, we assessed the expression of platelet EC adhesion molecule‐1 (CD31) and endomucin (EMCN) using immunofluorescence staining. The results revealed that the Pattern + CS group significantly promoted vessel formation, with most vessels exhibiting a type‐H vascular phenotype characterized by high expression of both CD31 and EMCN, as indicated by the white arrows in Figure 7J. The Bio‐Gide membrane also demonstrated angiogenic activity, but the majority of newly formed blood vessels exhibited a CD31^hi^ EMCN^low^ vascular phenotype. Quantitative analysis further confirmed that the BioLiving periosteum significantly enhanced the formation of CD31^hi^ EMCN^hi^ type‐H vessels (Figure 7K). It is interesting to note that the Pattern group markedly promoted bone formation while exerting minimal effects on angiogenesis. In contrast, when mini‐tissues were included (the Pattern + CS group), enhanced neovascularization was observed. This indicates that the physical and chemical signals exert distinct yet complementary functions, respectively promoting new bone formation and neovascularization. Therefore, the BioLiving periosteum was capable of promoting type‐H vessel angiogenesis, which was probably attributed to the type‐H ECs formed through the LSC method, as revealed in Figure 2.

Collectively, the physicochemical cues derived from the BioLiving periosteum effectively orchestrate and promote robust bone formation via type‐H vessel‐coupled osteogenesis in vivo.

### 2.8 The BioLiving Periosteum Triggers Centripetal Regeneration in Challenging Vertical Bone Augmentation

**FIGURE 8:**
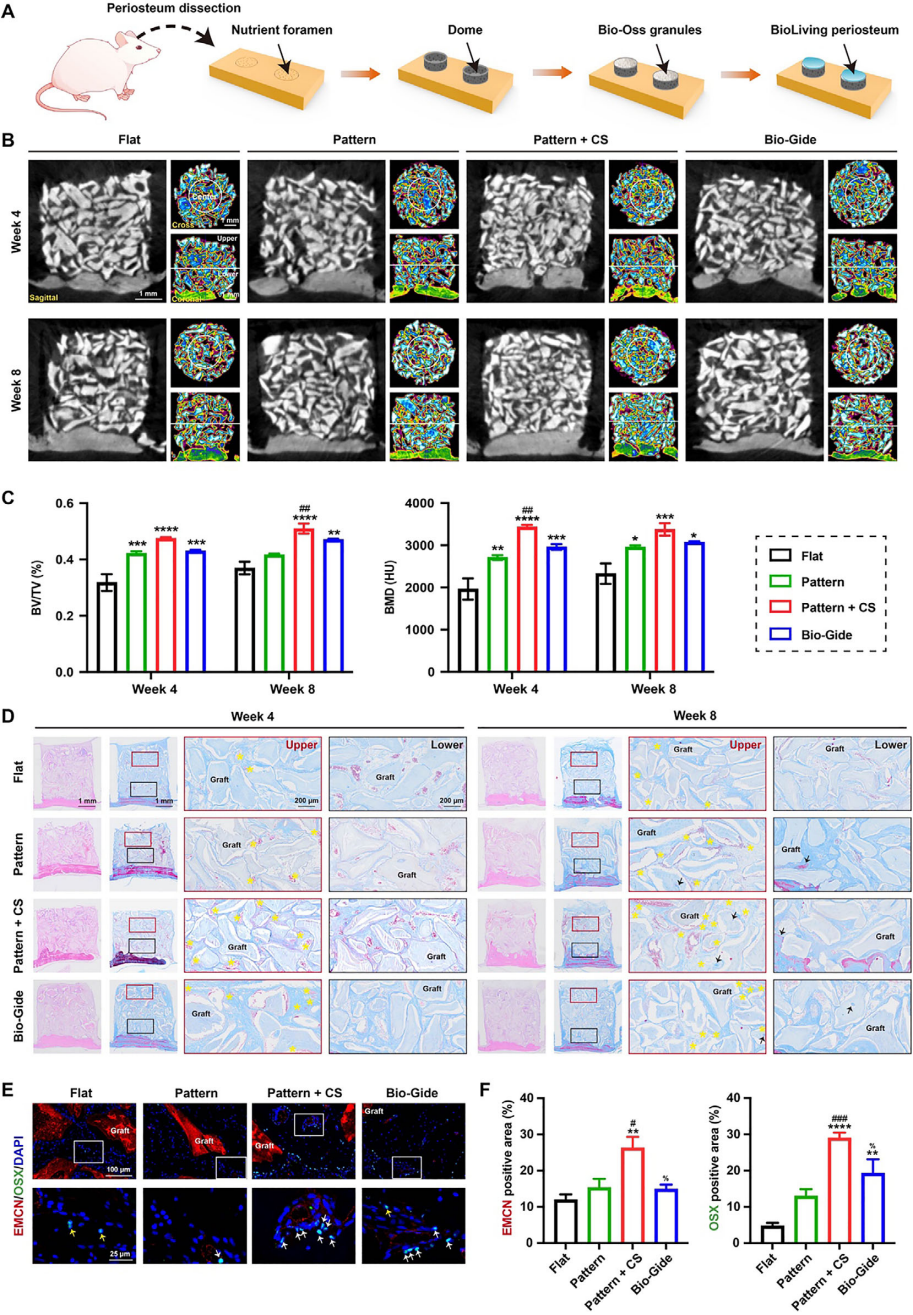
In vivo therapeutic performance of the BioLiving periosteum in vertical bone augmentation. (A) Schematic diagram of the vertical bone augmentation surgery process, and periosteum implantation. (B) Micro‐CT images in sagittal section and 2D pseudocolor micro‐CT images in cross and coronal sections of augmented areas at 4 and 8 weeks after treatment. (C) Quantitative analysis of bone volume/total volume (BV/TV) and bone mineral density (BMD) (*n* ≥ 5). (D) H&E staining and Masson's trichrome staining of augmented areas after 4 and 8 weeks of treatment (the yellow asterisks indicated neo‐vessels; the blank arrows indicated newly formed osteoids). (E) Representative images of multi‐immunofluorescence staining for type‐H vessels and OSX^+^ cells grown in the augmented areas (the white arrows indicated OSX^+^ cells surrounding the type‐H vessels; the yellow arrows indicated OSX^+^ cells located far from the type‐H vessels), and (F) the semiquantitative analysis of EMCN and OSX (*n* = 5). All statistical data are represented as mean ± SEM. Statistical analyses were performed using one‐way ANOVA with Tukey's post hoc test. ^*^ *p* < 0.05, ^**^ *p* < 0.01, ^***^ *p* < 0.001, and ^****^ *p* < 0.0001 compared with the Flat group. ^#^ *p* < 0.05, ^##^ *p* < 0.01, and ^###^ *p* < 0.001 compared with the Pattern group. ^%^ *p* < 0.05 compared with the Pattern + CS group.

In clinical dentistry, extensive bone augmentation is often required not only in major bone defects (e.g., due to tumors), but also in cases of alveolar ridge deficiency prior to dental implant placement. Such augmentation is particularly challenging due to the lack of surrounding blood supply, compared to conventional large‐area bone grafting. Therefore, in this study, we further established a vertical bone augmentation model to simulate this more demanding scenario of large bone defect reconstruction. To evaluate whether the designed BioLiving periosteum could support rapid vascularization and efficient osteogenesis in such challenging circumstances, a vertical bone augmentation model on calvarial bone in rats was established. The surgical procedures of vertical bone augmentation and periosteum implantation were illustrated in Figure 8A. Briefly, after creating a 5‐mm bone ring via osteotomy technique, several nutrient foramina were drilled to allow blood infiltration. A PCL dome was placed to fit the osteotomy line, creating a stable vertical bone augmentation space. The periosteum from different groups was then placed above after commercial xenogenic bone substitutes were filled into the augmented area.

At weeks 4 and 8 post‐implantation, the animals were sacrificed, and the overall bone repair level was evaluated both qualitatively and quantitatively using micro‐CT. Since the high density of Bio‐Oss granules in X‐ray images made the regenerated bone difficult to detect, 2D pseudocolor images with cross and coronal sections were used to present visible new bone formation (Figure 8B). Neo‐bone formed in relatively low density (yellow‐green), surrounding the denser Bio‐Oss granules (brilliant blue), while fibrous tissue appeared in purple with the lowest density. Notably, under the guidance of the BioLiving periosteum, the central part of the augmented area was filled with a greater amount of neo‐bone compared with other groups, with the yellow‐green color being more evident in the central region (as indicated by the central white ring). Particularly, a significantly larger amount of neo‐bone formed in the upper part of the augmented area in the Pattern + CS group (as shown in the coronal view), despite its distance from the nutrient foramen, which was typically associated with limited regenerative cellular sources and blood vessel infiltration. In contrast, only a limited amount of new bone formed in the upper area of the other groups, even after 8 weeks. The quantitative results further revealed that the BioLiving periosteum treatment induced the highest BV/TV and BMD. However, the significant difference was somewhat underestimated due to the presence of high‐density Bio‐Oss granules (Figure 8C).

To further visualize the microscopic details of the augmented areas, histological examinations were performed (Figure 8D). After 4 weeks of implantation, fibrous tissue was observed surrounding the Bio‐Oss grafts, within which varying numbers of blood vessels could be detected. As expected, with effective blood vessel infiltration from the drilled nutrient foramen, the lower part of the augmented area exhibited a higher density of blood vessels compared to the upper section. Notably, the BioLiving periosteum induced a more abundant presence of neo‐vessels in the upper part (as indicated by the yellow asterisks), suggesting that, in addition to the nutrient foramen, the BioLiving periosteum could also promote rapid vascularization to replenish the upper area with a nutrient supply. By 8 weeks, osteoids began to form within both the fibrous tissues and the Bio‐Oss grafts, as indicated by the black arrows. Moreover, the implantation of the BioLiving periosteum enhanced osteoid deposition in the upper part and mineralization of newly formed bone at the lower host bone border.

Immunofluorescence analyses were further conducted to assess type‐H vessel formation at 4 weeks (Figure 8E). As expected, a greater number of CD31^hi^ EMCN^hi^ blood vessels were detected in the Pattern + CS group, indicating the strong capacity of the BioLiving periosteum to induce type‐H blood vessel formation. This finding is in agreement with the aforementioned results (Figure 7J, K). Particularly, most OSX^+^ osteoprogenitor cells were surrounded by type‐H blood vessels, as indicated by the white arrows; this was also reported in previous studies. Kusumbe et al. found that over 70% of OSX^+^ cells were selectively positioned around type‐H endothelium because these ECs secrete many growth factors to recruit and stimulate the proliferation and differentiation of osteoprogenitors, thereby enhancing osteogenesis [8, 53]. Further, the number of OSX^+^ osteoprogenitors surrounding type‐H vessels was significantly increased in the Pattern + CS group, as confirmed by the quantitative analysis (Figure 8F). In comparison, only limited numbers of OSX^+^ cells were scattered in the regenerative tissue (indicated by yellow arrows), especially in the Flat group. However, the origin of the recruited OSX^+^ cells remains unknown. Whether both periosteal progenitors and bone marrow‑derived progenitors are mobilized by the BioLiving periosteum should be further investigated in subsequent studies. These results implied that the BioLiving periosteum had a strong effect on facilitating the formation of type‐H blood vessels, which in turn recruited osteoprogenitor cells and further accelerated bone formation in a centripetal regeneration mode.

Taken together, these data demonstrate that the type‐H ECs within the BioLiving periosteum effectively promote type‐H endothelium formation in vivo. This serves as a powerful driver for rapid vascularization and recruitment of progenitor cells, particularly in areas distant from the host tissue. Once recruited to the defect site, these progenitors are further guided by the topological patterns of the periosteum. These biochemical signals and biophysical cues collectively evoke an effective centripetal regeneration mode in challenging vertical bone augmentation.

## 3 Conclusion

Inspired by the topological structure and multiple biofunctions of natural periosteum, a BioLiving periosteum containing cell‐sheet‐like mini‐tissue was developed to address the high biological demands of large bone defects. Particularly, the LSC method endowed ECs with a type‐H phenotype by driving their active glycolytic metabolism. This, in turn, enabled their secretion of a series of angiogenic and osteogenic biomolecules and the formation of functional type‐H vessels. OSX^+^ osteoprogenitors were initially recruited by these biochemical cues released from type‐H endothelium and then guided by the topological cues from the patterned hydrogel for central migration and directional arrangement. The combination of remote biochemical guidance and contact biophysical guidance orchestrated angiogenesis‐osteogenesis coupling, thereby evoking efficient centripetal bone formation in vertical bone augmentation. Therefore, this BioLiving periosteum mitigates the challenges associated with insufficient blood supply and cellular sources, enhances effective bone regeneration by replicating the natural biofunction of the periosteum, and offers a promising strategy for addressing challenging bone defects.

## 4 Experimental Section

### 4.1 Fabrication of the BioLiving Periosteum

GelMA was purchased from EFL and characterized by our previous study [29]. GelMA prepolymer solution (10% (w/v)) was prepared by dissolving the freeze‐dried GelMA (EFL‐GM30 or EFL‐GM60, purity > 99.9%) in phosphate buffered saline (PBS) at 37°C and adding lithium phenyl‐2,4,6‐trimethylbenzoylphosphinate (LAP, 0.5% (w/v), purity > 99.8%). Then, dopamine (DOPA, 0.5% (w/v) or 1% (w/v)) and sodium alginate (0.5% (w/v) or 0.8% (w/v)) were added to the ink, respectively. After being fully shaken and dissolved, the ink was filtered through a 0.22‐µm filter membrane for sterilization. Resin molds with aligned and radial patterns were designed by Solidworks and fabricated by a high precision 3D printer (nanoArch S140). The aligned resin mold featured a line width of 50 µm and a line height of 50 µm. The radial resin mold had a radial line width that varied from 40 µm to 80 µm, increasing from the inside to the outside, with a consistent line height of 50 µm. Then, PDMF molds with aligned and radial patterns were obtained by the mold flipping method. The prepared GelMA ink was dropped onto the patterned mold and photo‐crossed with a UV light (405 nm, 5 mW cm^−2^) for 200 s. After being fully crosslinked, the patterned hydrogels were demolded and sterilized overnight by the UV light.

### 4.2 Adhesion Characterization of the Composite Hydrogels

Shear adhesion test of GelMA with different content of dopamine was performed. Briefly, a 10 × 10 mm composite hydrogel was crosslinked and alternately adhered to two 10 × 30 mm glass slide and the other side of the glass slide was used for the adhesion test. During the test, the prepared glass slides were clamped securely in the testing grips, with a fixed gauge length of 10  mm with a constant 10 N force to establish a good contact with each other. Then, the two glass slides were pulled in parallel to the glass surface at a constant rate of 1 mm min^−1^ to measure the shear tissue adhesion strength.

### 4.3 Mechanical Characterization of the Composite Hydrogels

The mechanical properties of GelMA contained with dopamine and sodium alginate were examined. The casted samples were fabricated with the following steps. Briefly, (i) The tip of a 3 mL syringe was cut off and the composite prepolymer (0.5 mL) was added to the adapted syringe. A UV light was used to fully crosslink the hydrogel. (ii) Then, the hydrogel was carefully pushed out of the syringe by the piston. For the compression test, samples were placed at the center of a couple of flat probes. The loading rate was set as 0.2 mm min^−1^, and stopped when the stress dropped over 50% within one sampling step. The stress–strain curve was automatically recorded by the testing system. The compression strength was chosen as the largest stress before failure, while the compression modulus was calculated by the stress and strain within the elastic zone (0%–10% strain).

### 4.4 Swelling Property Test

The swelling properties of the composites were evaluated by measuring dimensions. Initially, all GelMA composite hydrogels were casted with a diameter of 5 mm (Di). After immerged in sterile PBS at 37°C for 0, 2, 6, 12, and 24 h, the composite hydrogels were taken out. Excess liquid was removed using wax paper, and the diameters of samples were measured again. The final diameter of the weighed sample (Df) was recorded. The mass SR was defined by the following equation.

$$\mathrm{SR}\;=\frac{\mathrm{Df}-\mathrm{Di}}{\mathrm{Di}}\;\times 100\%$$

### 4.5 Biodegradation Rate Test

The hydrogel samples in GM (GelMA) and GAD (GelMA incorporated with sodium alginate and DOPA) groups were treated in 0.002 U mL^−1^ collagenase II PBS solution. At each time point, samples were collected and measured the residual mass. In the meanwhile, the collagenase II PBS solution was refreshed.

### 4.6 Structural Characterization of the Composite Hydrogels

To characterize the structure of the composite hydrogels, hydrogels with different components were solidified under a UV light. After being lyophilized for 48 h, the dried samples were coated with platinum in a sputter coater (Ion Sputter E‐1045, Hitachi, Japan), and imaged with SEM (SU‐6600, Hitachi). FTIR (IRAffinity‐1, Shimadzu, Japan) was performed with 30 scans at 4 cm^−1^ resolution from 4000 to 400 cm^−1^. The background was determined using blank KBr plates.

### 4.7 Cell Culture

BMSCs were isolated from Sprague Dawley rats (aged 3 weeks) and purified by passaging in 𝛼‐minimum essential medium (𝛼‐MEM, HyClone, USA) with 10% fetal bovine serum (Gibco, USA) in a 5% CO_2_ incubator at 37°C. After being cultured for 3–5 generations, the cells were used in the subsequent experiment. The HGF‐1 cells and HUVECs (ATCC, USA) were cultured in Dulbecco's modified Eagle medium (DMEM, HyClone) containing 10% fetal bovine serum. For 2D cell culture, cells were cultured on the traditional culture plate and the culture medium was changed every 48 h. For 3D cell culture, the LSC method was used according to our previous work. The fluorinated oil (3M Novec 7500 Engineered Fluid) or PDMS (Sylgard 184, DOW Chemical Company) was sterilized overnight by the UV light and preadded to the culture plate as a liquid substrate. The amount of suitable fluorinated oil used was corelated with the area of the culture chamber. Specifically, for commercial 24‐well plates, 1 mL of fluorinated oil per well was appropriate. The cells were cultured in a humidified atmosphere of 95% air and 5% CO_2_ at 37°C and the culture medium was changed every 48 h.

### 4.8 RNA‐Seq Analysis

RNA‐Seq was performed using Illumina HiSeqTM 4000 platform (Tsingke, BeijingChina). Briefly, total RNA was extracted from HUVECs cultured in either 2D or 3D environment using the Trizol reagent kit (15596, Invitrogen, USA). RNA quality was assessed on an Agilent 2100 Bioanalyzer (Agilent Technologies, USA) and checked using RNase free agarose gel electrophoresis. Then the cDNA fragments were purified with QiaQuick PCR extraction kit (Qiagen, The Netherlands), end repaired, poly(A) added, and ligated to Illumina sequencing adapters. DEGs were identified using DESeq2 software, and the genes with the parameter of *p*‐value < 0.05 and absolute fold change > 1.5 were considered DEGs. Heatmaps were generated using the pheatmap package. Correlation heatmaps were generated using the pheatmap package. Dynamic time‐series expression patterns generated using the Mfuzz package. GO and KEGG pathway enrichment analysis of DEGs was performed to screen the significantly enriched terms using R (v3.2.0), respectively. GSEA was performed using irGSEA software.

### 4.9 Metabonomics

Metabonomics was performed using LC‐MS (LC Bio, Hangzhou, China). The collected supernatants of HUVECs cultured in either 2D or 3D environment were thawed on ice, and metabolite were extracted with 80% methanol Buffer. Briefly, 50 mg of sample was extracted with 0.5 mL of precooled 80% methanol. The extraction mixture was then stored in 30 min at −20°C. After centrifugation at 20,000 g for 15 min, the supernatants were transferred into new tube to and vacuum dried. The samples were redissolved with 100 µL 80% methanol and stored at −80°C prior to the LC‐MS analysis (Vanquish Flex UHPLC (Thermo), Q‐Exactive (Thermo)). In addition, pooled QC samples were also prepared by combining 10 µL of each extraction mixture. Statistical analysis was performed in R (version 4.0.0). The raw protein intensity will be normalized by method “medium”. Hierarchical clustering was performed using the pheatmap package. The PLSDA analysis is performed by the R package ropls and the VIP values of each variable are calculated. Correlation analysis was performed by Pearson correlation coefficient of cor package. The three conditions of *p*‐value < 0.05, difference multiple > 1.2 obtained by T test and VIP calculated by PLSDA analysis simultaneously met the screening of the final metabolites with significant differences. Hypergeometric‐based enrichment analysis with KEGG Pathway was performed to annotate protein sequences. The software GSEA (v4.1.0) and MSigDB were used for GSEA to determine whether a set of genes in a specific KEGG pathway in different situations. Meeting this condition |NES| > 1, NOM *p*‐value < 0.05, FDR *q*‐val < 0.25 were significantly different between the two groups. Bioinformatic analysis and metabolic network construction were performed using the OmicStudio platform (https://www.omicstudio.cn/tool), which generated pathway‐specific network maps by integrating metabolite‐KEGG pathway associations.

### 4.10 RT‐PCR Assay

Total mRNA was extracted from the cells by TRIzol reagent (15596, Invitrogen) and reverse‐transcribed to cDNA using PrimeScript RT Master Mix (RR036, Takara, Japan). The quantitative polymerase chain reaction was performed by TB Green Premix Ex Taq (RR420A, Takara) on a CFX384 Real‐Time PCR System (CFX384 Touch, Bio‐Rad, USA). The quantitative polymerase chain reaction program was conducted with initial denaturation (95°C for 30 s) followed by 40 cycles of denaturation (95°C for 5 s) and annealing/extension (60°C for 32 s). The mRNA abundances of target genes were normalized to GAPDH and calculated via the standard 2^−△△Ct^ method. The sequences of the primers used in this study are listed in Table S1.

### 4.11 Western Blot

Cells were first lysed with RIPA lysis buffer. Equal amounts of proteins were separated by SDS‐polyacrylamide gel electrophoresis gel in 1×running buffer (25 mm Tris, 192 mm glycine, 1% SDS), transferred to polyvinylidene difluoride membranes in 1×transfer buffer (25 mm Tris, 192 mm glycine, 20% methanol) were used in Western blotting, and incubated with primary antibodies according to the manufacturers’ instructions. Primary antibodies were detected by an appropriate horseradish peroxidase‐conjugated secondary antibody and exposed with Immobilon Western Chemiluminescent Horseradish Peroxidase Substrate. Blotting signal intensities were quantified using Image Lab software. The detailed information on antibodies used in this study is listed in Table S2.

### 4.12 Enzyme‐Linked Immunosorbent Assay

To measure the cytokines secreted by cells in different groups, the corresponding supernatants were collected at 1, 2, and 3 days. The secretion of cytokines, including VEGF (Bioswamp, HM10057), TGF‐β (Bioswamp, HM10058), aFGF (Bioswamp, HM10408), and PDGF (Bioswamp, HM10054), was then measured by commercial ELISA kits following the manufacturer's guidance.

### 4.13 Morphological Analysis of Cells

To visualize the arrangement behavior of cells in hydrogels, BMSCs and fibroblasts were seeded either on the flat or patterned hydrogel disks, respectively, for 3 days. After fixation with 4% paraformaldehyde for 30 min, all disks were permeabilized with 0.5% Triton X‐100 for 15 min. Then, the samples were stained with FITC phalloidin (C8001, Bioss) for 30 min. From this point onward, the samples were strictly protected from light. DAPI (D9542, Sigma, USA) was then added for 10 min. Finally, cells were observed with a laser scanning confocal microscope (LSM780, ZEISS, Germany). To visualize the morphology of cells cultured in 2D and 3D culture environments, cells were seeded either on a traditional culture plate or the liquid substrate for 1 day. The samples were first fixed with 2.5% glutaraldehyde in phosphate buffer (0.1 m, pH 7.0) for more than 4 h. Then the samples were postfixed with 1% OsO4 in phosphate buffer for 1–2 h. After double fixation, the samples were dehydrated by a graded series of ethanol (30%, 50%, 70%, 80%, 90%, and 95%) for about 15 min at each step, then dehydrated twice by alcohol for 20 min at each step. After that, the samples were dehydrated in a critical point dryer (Model HCP‐2, Hitachi). Finally, the samples were coated with gold–platinum and imaged with SEM (SU‐6600, Hitachi).

### 4.14 Cell Infiltration Assay

To observe the fibroblast infiltration behavior, the fibroblasts and BMSCs were firstly stained with DiD (C1039, Beyotime, yellow) and DiL (C1036, Beyotime, red), respectively, according to the manufacturer's guidance. Then the dyed cells and GFP‐labeled ECs were seeded either on the upper chamber of the Transwell system(3460, Corning)or the liquid substrate. The infiltration behavior of fibroblasts was observed by a laser scanning confocal microscope (LSM780, ZEISS, Germany) and quantified by ImageJ software (NIH, USA). Specifically, the area of yellow fluorescence was used as an indicator to represent the number of fibroblasts that migrated from top to bottom. To ensure consistency across different time points, uniform image acquisition parameters and fluorescence intensity analysis thresholds were applied across all experimental groups. Detailed counting criteria, including the threshold setting for fluorescence signals and the method for selecting regions of interest, were set to ensure the reliability and comparability of the experimental results.

### 4.15 Cell Proliferation Analysis

The cytocompatibility of different hydrogels were measured using a cell counting Kit‐8 (CK04, Dojindo Chemical Technology, Japan). Cells were seeded in a 24‐well plate and cultured with conditioned medium extracted from different hydrogels. Then, the culture medium was discarded, and PBS was added to wash the cells three times. After that, a mixture of 1450 µL culture medium and 50 µL CCK‐8 reagent was added to each well. After 3 h of incubation, the mixture was transferred to a 96‐well plate to measure the optical density (OD) values at a wavelength of 450 nm.

### 4.16 Cell Viability Assays

The cell viability of the samples was measured after 3 days using a cell LIVE/DEAD assay. First, the samples were washed with PBS three times. Then, the samples were stained with a Calcein‐AM/PI staining kit (BB‐4126, BestBio, China). Live cells (Calcein‐AM^+^PI^−^) and dead cells (Calcein‐AM^−^PI^+^) were observed under a fluorescence‐inverted microscope (IX73, Olympus, Japan).

### 4.17 ALP Activity and ARS Staining

To evaluate the osteogenic potential of different hydrogels, BMSCs were seeded directly on the hydrogel disks and differentiated with medium containing 𝛽‐glycerophosphate (10 mM, G9422, Sigma), ascorbic acid (50 µg mL^−1^, A8960 Sigma), and dexamethasone (10^−8^ M, D4902, Sigma). For ALP staining, cultured cells were rinsed with PBS, fixed in 4% paraformaldehyde, and stained with a BCIP/NBT ALP color development kit (P0321, Beyotime, China). ALP activity was quantified using a LabAssay ALP kit (291‐58601, Wako Pure Chemical Industries, Japan). The total cellular protein content was determined using the BCA protein assay (P0010S, Beyotime), and the absorbance at 405 nm was recorded using a microplate reader (SpectraMax i3, Molecular Devices, USA). ARS staining was used to observe the formation of calcium nodules in BMSCs. To visualize calcification, cells were fixed in 4% paraformaldehyde and then stained with ARS for 15–30 min. For quantification, cells were washed three times with PBS and eluted with 10% cetylpyridinium chloride for 30 min, and absorbance was measured at 562 nm.

### 4.18 Establishment of Rat Calvarial Defect

A critical‐sized calvarial defect model was used to assess the in vivo bone formation capacity. The animal study was approved by the Ethics Committee for Animal Research at Zhejiang University (ethics approval number: ZJU20241052). Briefly, a total of 60 male Sprague‐Dawley (SD) rats (8 weeks old, 250–300 g) were divided into five groups: Blank, Flat, Pattern, Pattern + CS, and Bio‐Gide. After anesthetizing, shaving, and disinfecting the rats, an incision was made at the surgical site to expose the calvarium. After removing the natural periosteum, two symmetrical calvarial defects with a diameter of 5 mm were created on each sides of the cranial bone. Then the periosteum in different groups was used to fill the defect area, and the wound was sutured. Postoperative antibiotic prophylaxis was administered for 3 days to prevent surgical site infection.

### 4.19 Establishment of Vertical Bone Augmentation Model

A vertical bone augmentation model was further used to assess the angiogenesis and osteogenesis capacity of different periosteum in challenging bone defects. Briefly, a total of 48 male SD rats (8 weeks old, 250–300 g) were divided into four groups: Flat, Pattern, Pattern + CS, and Bio‐Gide. After creating a 5‐mm bone ring via osteotomy technique, multiple nutrient foramens were meticulously drilled within the circle to facilitate blood vessel infiltration. A 3D printed polycaprolactone (PCL) dome scaffold was subsequently positioned to fit the osteotomy margin, thereby establishing a stable three‐dimensional space for vertical bone augmentation. After filling the augmented compartment with commercially available xenogenic bone graft substitutes (Geistlich Bio‐Oss), the periosteum in different groups was carefully covered above. Then, the wound was sutured and postoperative antibiotic prophylaxis was administered for 3 days to prevent surgical site infection.

### 4.20 Micro‐CT and Histological Analysis

At 4‐ and 8‐weeks post‐implantation, the specimens were harvested and fixed in 4% paraformaldehyde for 24 h. Regions of interest (ROIs) were defined within the surgical area, and then the BV/TV and BMD of each sample were calculated from the selected ROI (Skyscan 1276, Bruker, Germany). After micro‐CT analysis, the samples were decalcified by soaking in decalcifying solution at 37°C. The PCL dome was removed after decalcification. Then, the samples were dehydrated in a gradient alcohol, embedded in paraffin, and sectioned into 5‐µm slices. The slices were treated with H&E and Masson's trichrome staining to assess new bone formation at 4 and 8 weeks.

### 4.21 Immunohistochemistry and Immunofluorescence Analysis

OSX and ALP expressions within the defect regions were tested by immunohistochemistry with antibodies. The histological and immunohistochemistry images were taken using a microscope (Olympus). Immunofluorescence staining of CD31 and EMCN, as well as EMCN and OSX was additionally performed to evaluate the new blood vessel formation. The detailed information on antibodies is listed in Table S2. ImageJ software (NIH) was employed to quantify the positively stained cells.

### 4.22 Statistical Analysis

Statistical analyses were performed with GraphPad Prism 8.0 or Origin 2022 software. Shapiro–Wilk test (*p* < 0.05) was used to test normality. All data passed the normality test and were plotted as the means ± SEMs. Each experiment was performed with a minimum of three biological replicates (*n*); exact numbers are mentioned in the related figure legends. For comparisons between two groups, an unpaired two‐tailed Student's *t*‐test was used. For comparisons between multiple groups, analysis of variance (ANOVA) with a multiple comparison follow‐up test was used. The statistical tests and *p* values were indicated in the related figure legends. *p* value of below 0.05 was considered significant.

## Author contributions

Y.S. and N.L. conceived the ideas for experimental designs. Y.S., N.L., and J.G. conducted most of the experiments, analyzed data, and prepared the manuscript. Y.S., Y.W., W.W., and Z.K. conducted animal experiments and analyzed data. N.L. and W.K. conducted characterization and analyzed data. M.X., W.J., and J.S. provided critical suggestions and instructions for the project and helped compose the manuscript. H.P., Y.H., and Z.X. developed the concept, supervised the project, and edited the manuscript.

## Conflicts of Interest

The authors declare no conflict of interest.

## Supporting information




**Supporting File**: advs74659‐sup‐0001‐SuppMat.docx.

## Data Availability

The data that support the findings of this study are available from the corresponding author upon reasonable request.
