# Supplementary material for: A BioLiving Periosteum Evokes Centripetal Regeneration in Challenging Bone Defects
Source: Adv Sci (Weinh). 2026 Mar 1;13(26):e18401. doi: 10.1002/advs.202518401 (PMC13159148; doi:10.1002/advs.202518401)
Supplement: Supplementary file 1 — Supporting File: advs74659‐sup‐0001‐SuppMat.docx. [file ADVS-13-e18401-s001.docx]

**Supporting Information**

**A BioLiving Periosteum Evokes Centripetal Regeneration in Challenging Bone Defects**

Yang Shi^1^, Nian Liu^2,3^, Jingyi Gu^1^, Yinling Wang^1^, Weiye Wang^1^, Zhiwei Ke^1^, Mingjun Xie^1^, Wenjing Jin^1^, Weicheng Kong^2,3^, Jue Shi^1^, Hui Pan^1*^, Yong He^2,3,4*^, Zhijian Xie^1*^

^1^ Stomatology Hospital, School of Stomatology, Zhejiang University School of Medicine, Zhejiang Provincial Clinical Research Center for Oral Diseases, Key Laboratory of Oral Biomedical Research of Zhejiang Province, Cancer Center of Zhejiang University, Zhejiang-Singapore International Joint Laboratory of Oral Bioengineering, Hangzhou 310000, China

^2^ State Key Laboratory of Fluid Power and Mechatronic Systems & Liangzhu Laboratory, School of Mechanical Engineering, Zhejiang University, Hangzhou 310027, China

^3^ Key Laboratory of 3D Printing Process and Equipment of Zhejiang Province, College of Mechanical Engineering, Zhejiang University, Hangzhou 310027, China

^4^ The Second Affiliated Hospital of Zhejiang University, Zhejiang University, Hangzhou 310027, China

*Corresponding author. Email: [xzj66@zju.edu.cn](mailto:xzj66@zju.edu.cn) (Z.X.); [yongqin@zju.edu.cn](mailto:yongqin@zju.edu.cn) (Y.H.); [7311020@zju.edu.cn](mailto:7311020@zju.edu.cn) (H.P.)

This PDF file includes:

Figures S1 to S10

Tables S1 and S2

**Supplementary Results:**


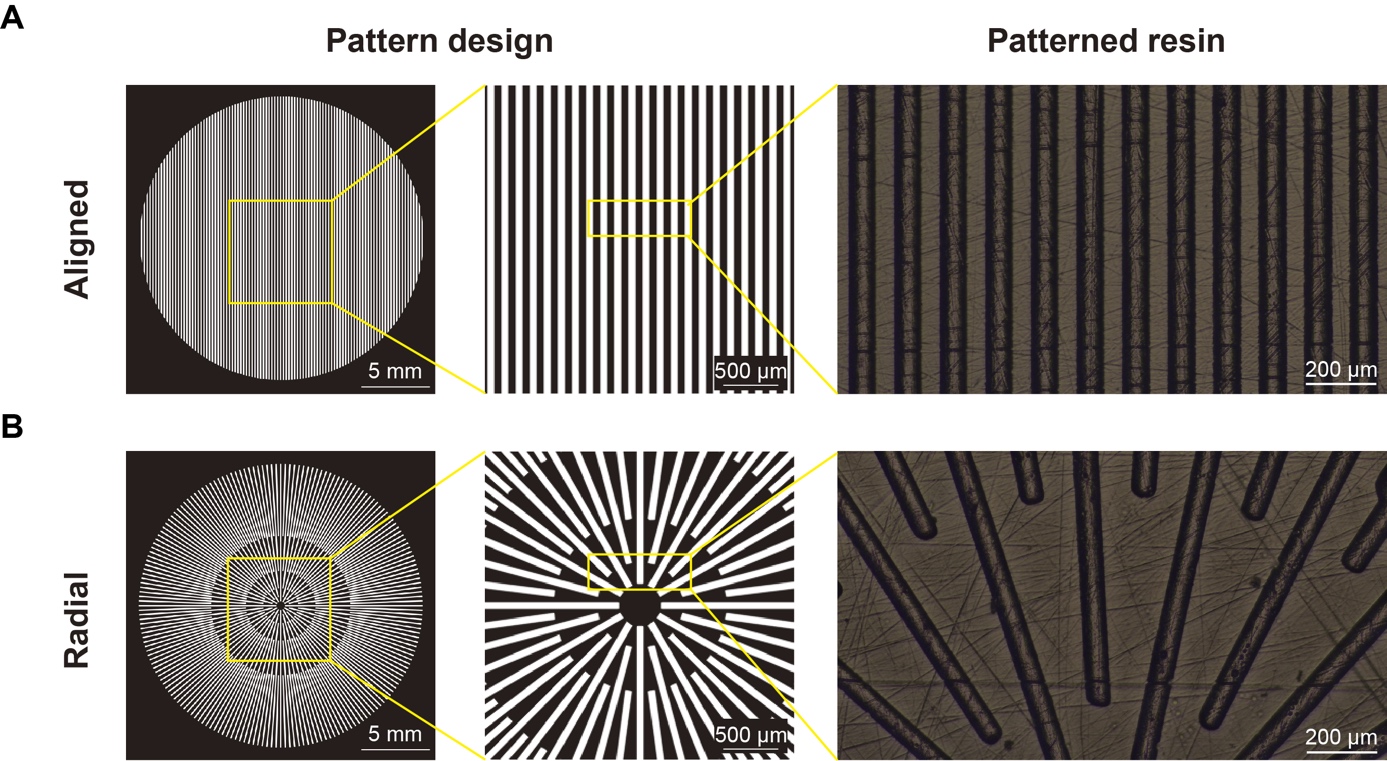


**Fig. S1.** A) Aligned pattern and B) radial pattern were designed by SolidWorks software, and the resultant resin templates were fabricated by the digital light process (DLP) 3D printing method.


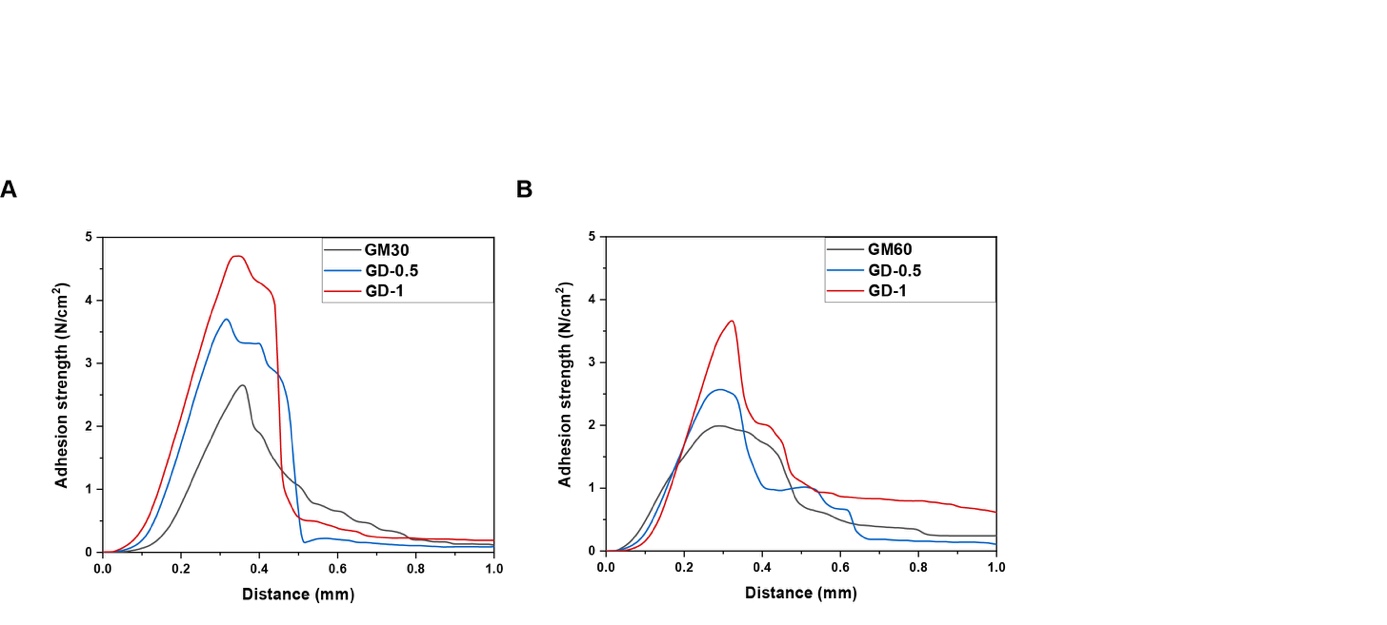


**Fig. S2.** The adhesion strength of A) GM30 and B) GM60 loaded with or without different content (0.5% and 1%) of DOPA.


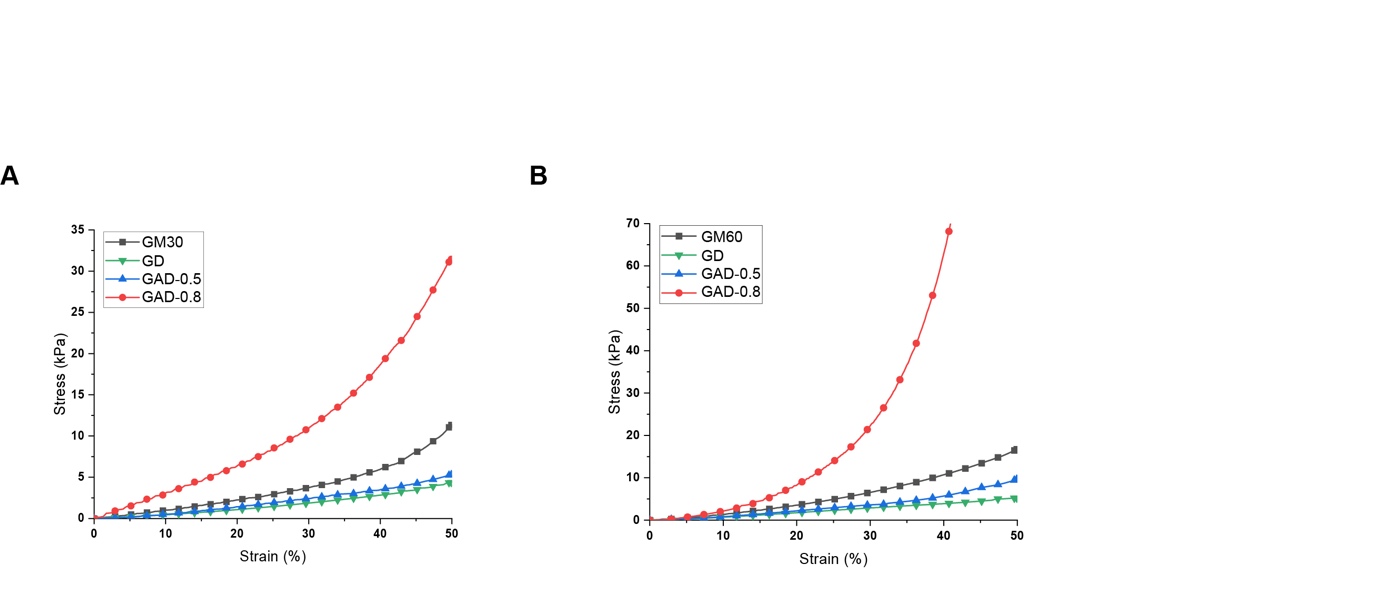


**Fig. S3.** The compressive strength of A) GM30 and B) GM60 loaded with or without 1% DOPA and different content (0.5% and 0.8%) of sodium alginate.


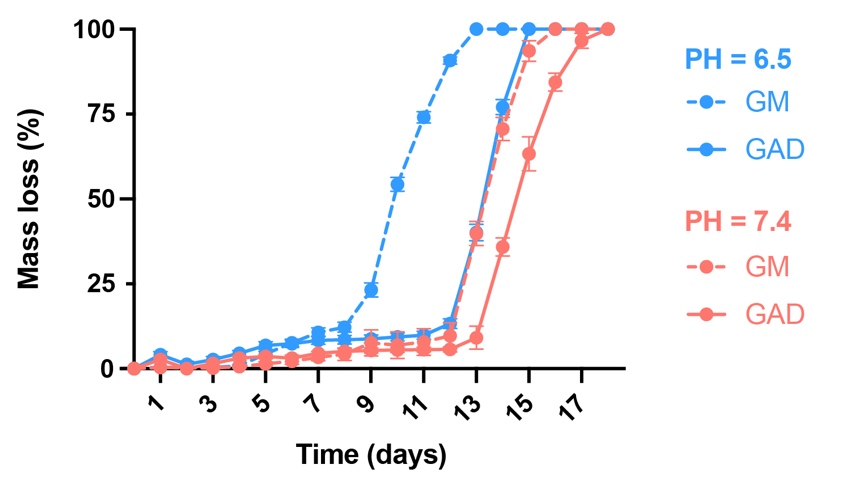


**Fig. S4.** Degradation profiles of GM (GelMA) and GAD (GelMA incorporated with sodium alginate and DOPA) hydrogels under different pH conditions (n ≥ 3). All statistical data are represented as mean ± SEM.


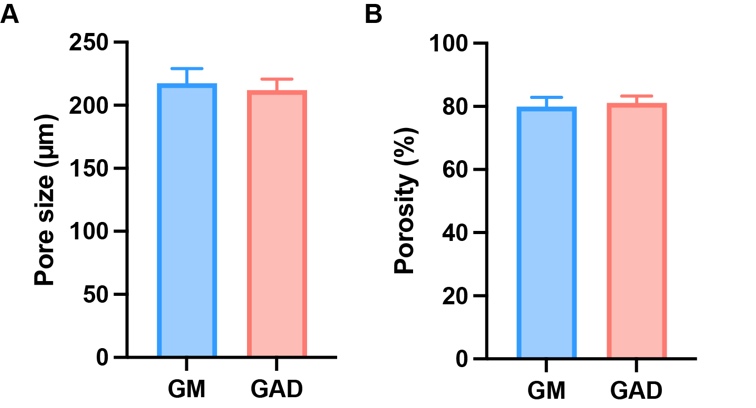


**Fig. S5.** Pore size (A) and porosity (B) of GM (GelMA) and GAD (GelMA incorporated with sodium alginate and DOPA) hydrogels. All statistical data are represented as mean ± SEM. Statistical analyses were performed using unpaired two-tailed Student's t-test.


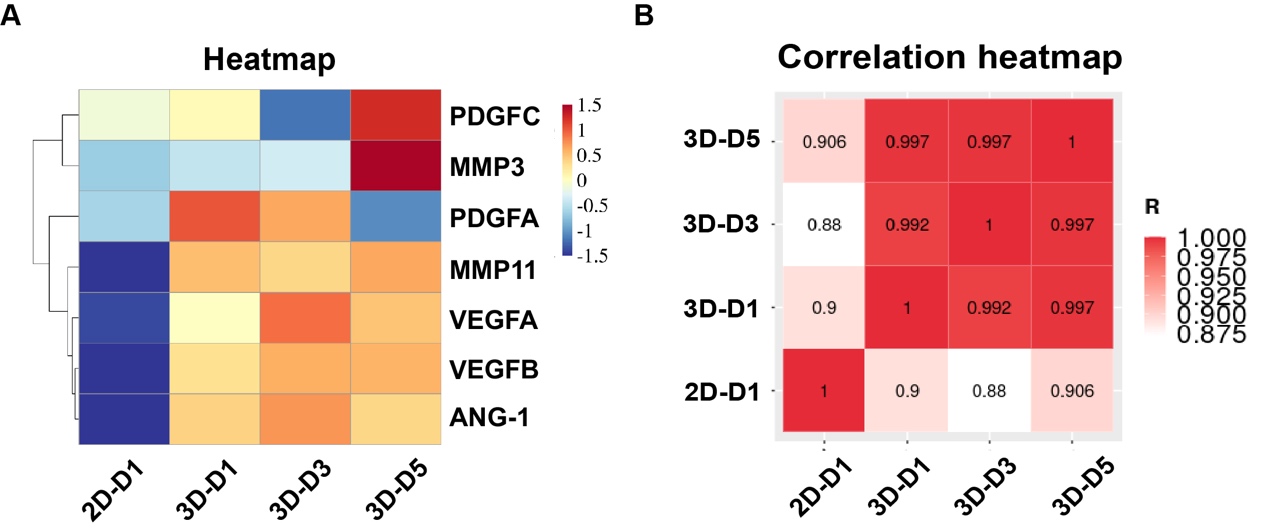


**Fig. S6.** A) Heatmap of angiogenesis-related genes in the 2D-cultured group on day 1 (2D-D1) and 3D-cultured groups on days 1, 3, and 5 (3D-D1, 3D-D3, and 3D-D5), and B) the corresponding Pearson’s correlation heatmap.


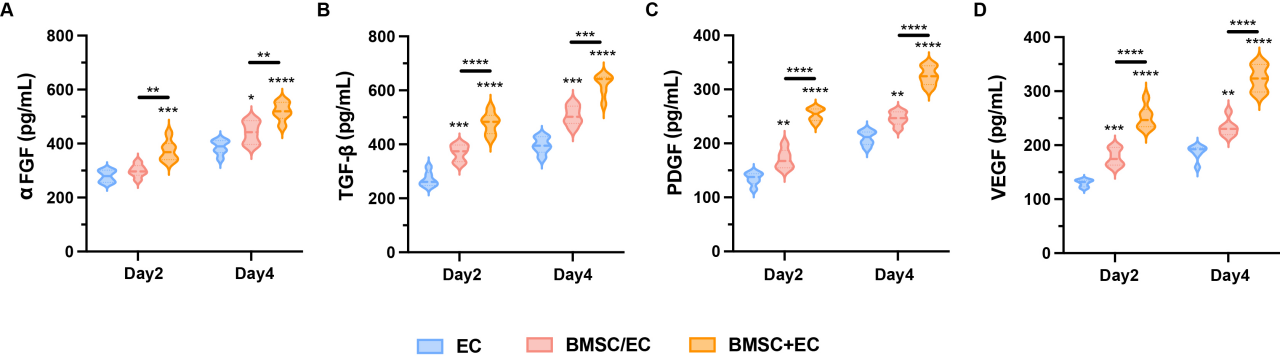


**F****ig. S7.** The concentrations of growth factors, including 𝛼FGF, TGF-β, PDGF, and VEGF, in the supernatant of ECs, BMSC/EC (ECs with BMSCs in the Transwell system), and BMSC+EC (ECs co-cultured with BMSCs) (n ≥ 3). ***p* < 0.01, ****p* < 0.001, and *****p* < 0.0001.


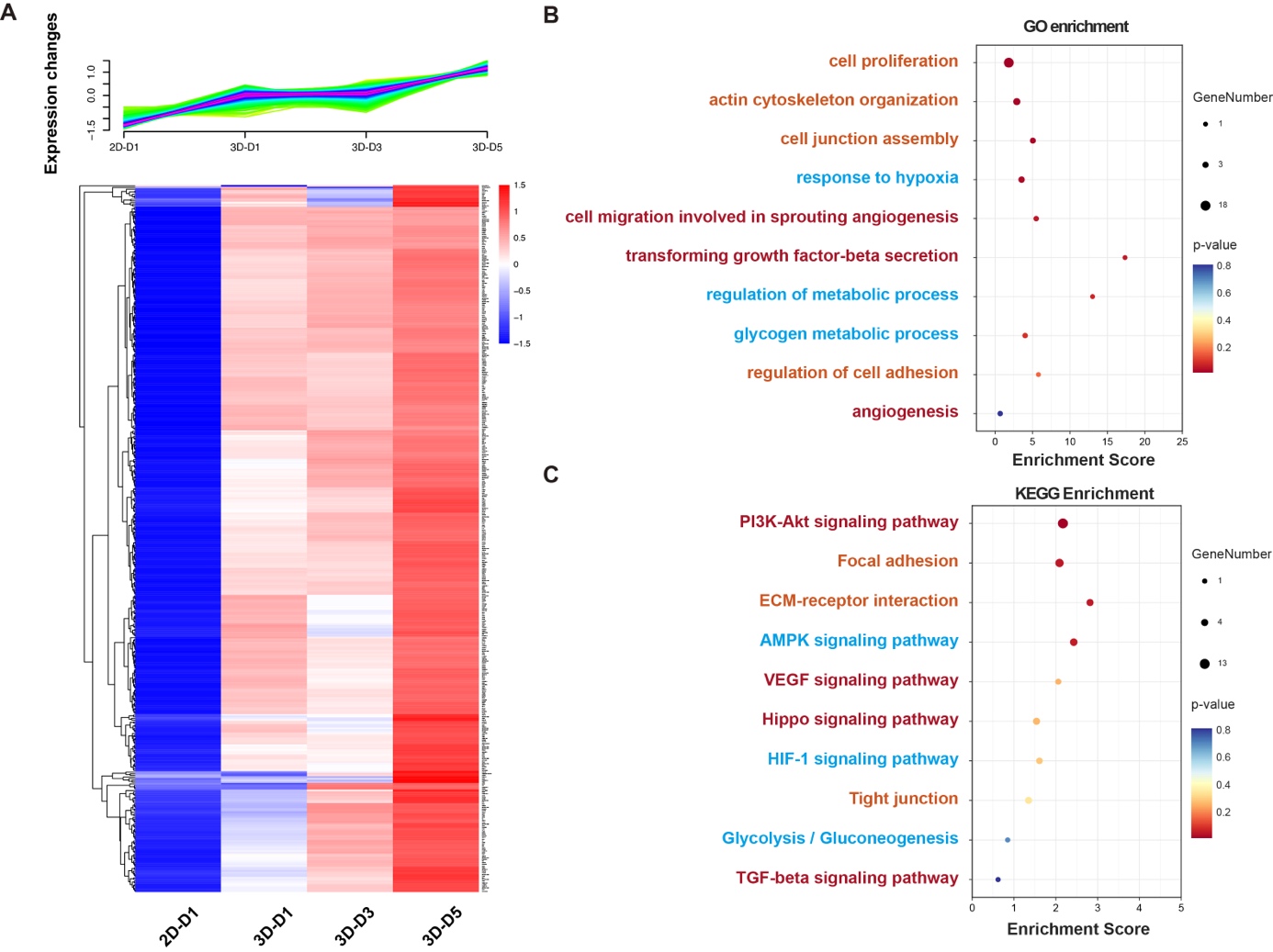


**Fig. S8.** Dynamic time-series expression patterns and pathway analysis. A) Heatmap of ECs in the 2D-cultured group on day 1 (2D-D1) and the 3D-cultured groups on days 1, 3, and 5 (3D-D1, 3D-D3 and 3D-D5), representing upregulated gene expression patterns over time. B) Gene Ontology (GO) and C) Kyoto Encyclopedia of Genes and Genomes (KEGG) enrichment analyses for the upregulated genes. (Yellow: cell junction and cytoskeleton-related terms; red: angiogenesis-related terms; blue: glycolysis-related terms)


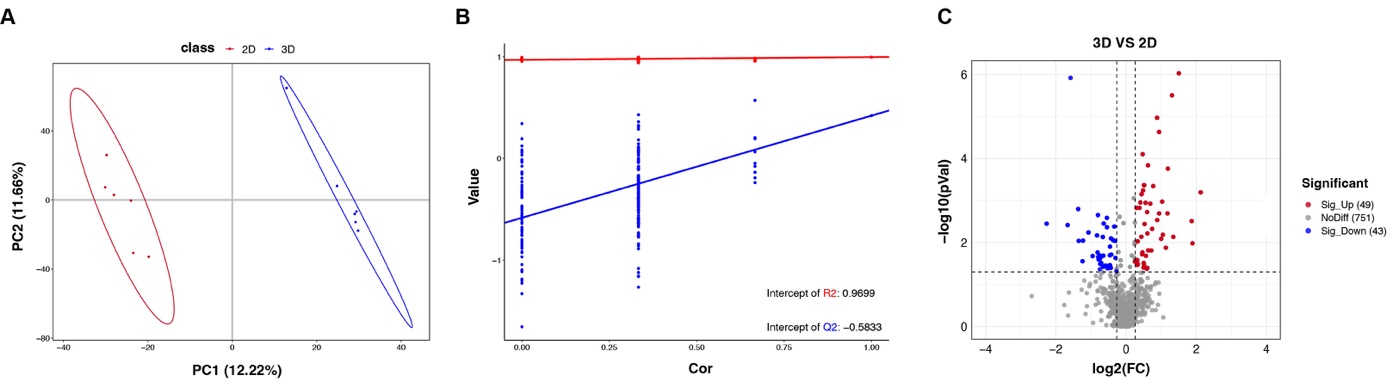


**Fig. S9.** A) Principal component analysis (PCA) of metabolomic profiles. B) Correlation analysis of significantly altered metabolites. C) Volcano plot map of differentially altered metabolites.


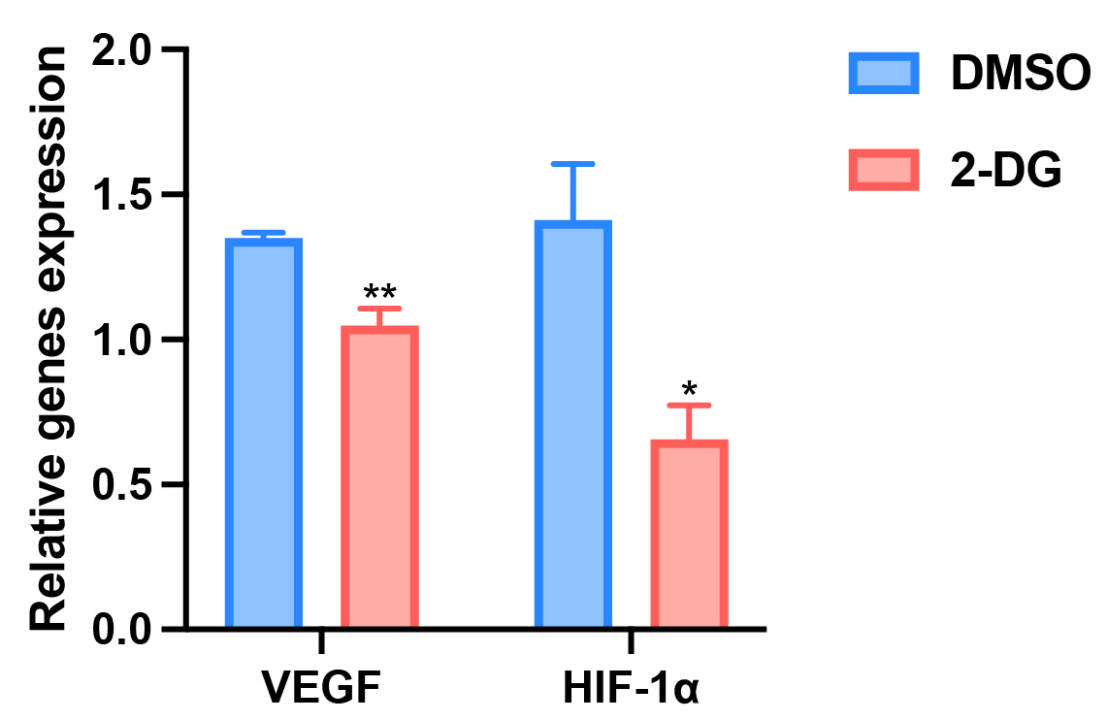


**Fig. S10.** The relative expression level of VEGF and HIF-1𝛼 in 3D-cultured ECs with or without 2-DG treatment (n ≥ 3). All statistical data are represented as mean ± SEM. **p* < 0.05 and ***p* < 0.01 compared with the DMSO group.


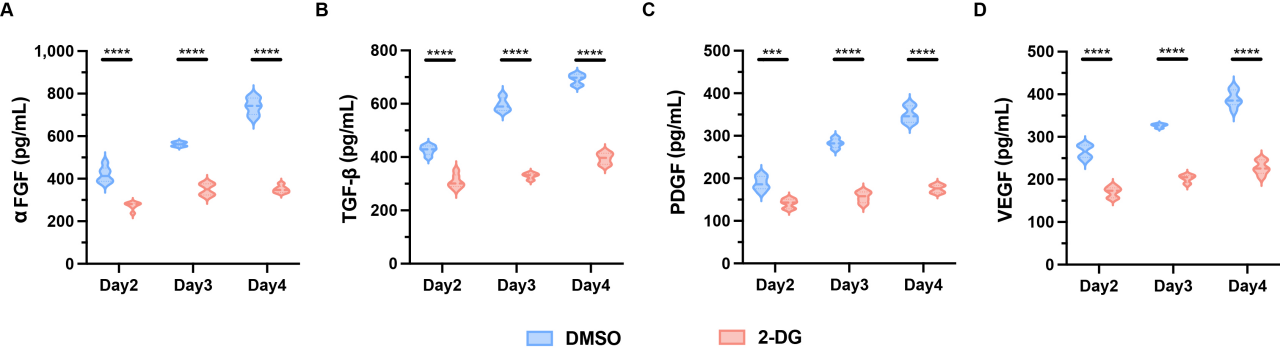


**Fig. S11.** The concentrations of growth factors, including 𝛼FGF, TGF-β, PDGF, and VEGF, in the supernatant of 3D-cultured ECs with or without 2-DG treatment on days 2, 3, and 4 (n ≥ 3). ****p* < 0.001 and *****P* < 0.0001 compared with the DMSO group.


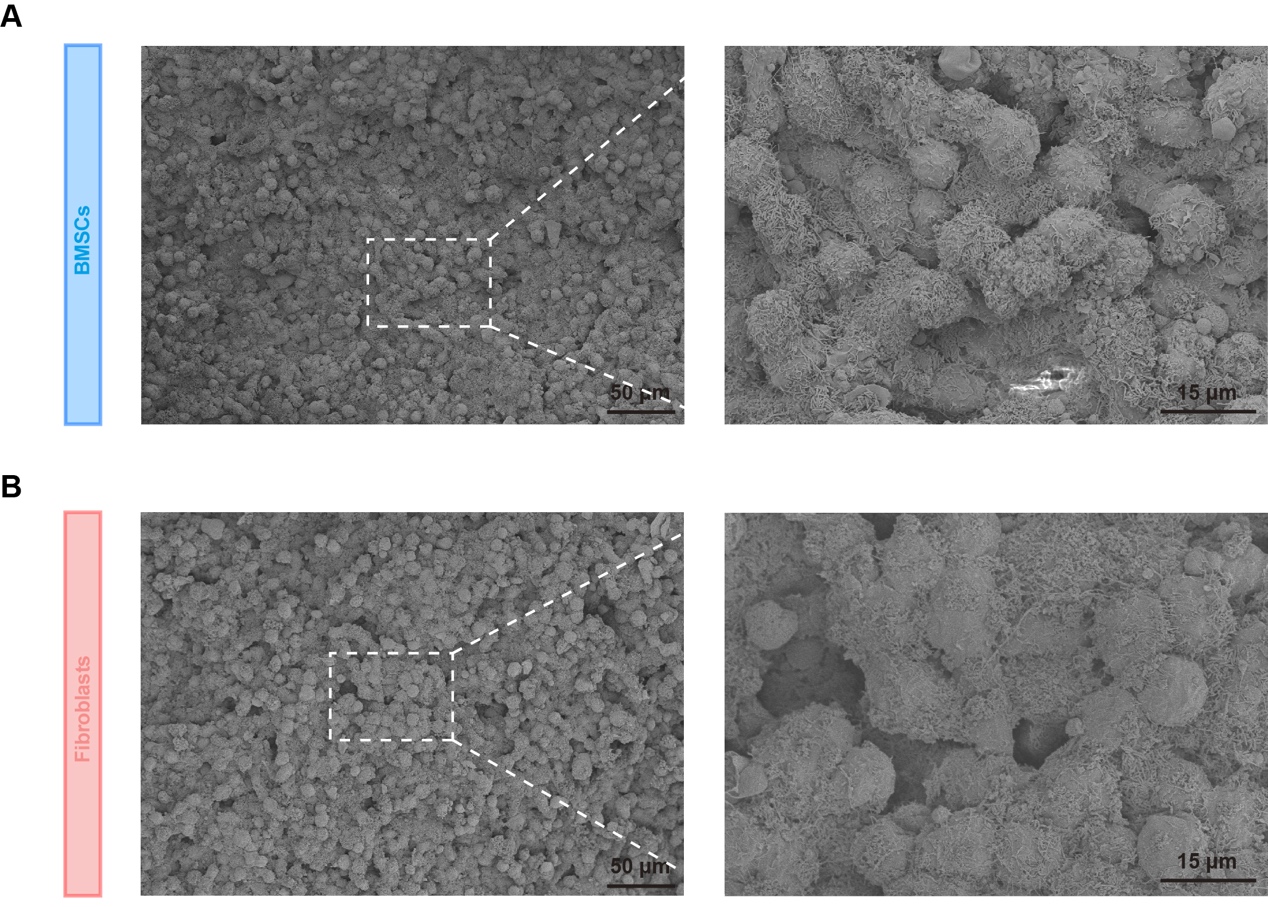


**Fig. S12.** SEM images of A) BMSCs and B) fibroblasts cultured with the LSC method at different magnifications.


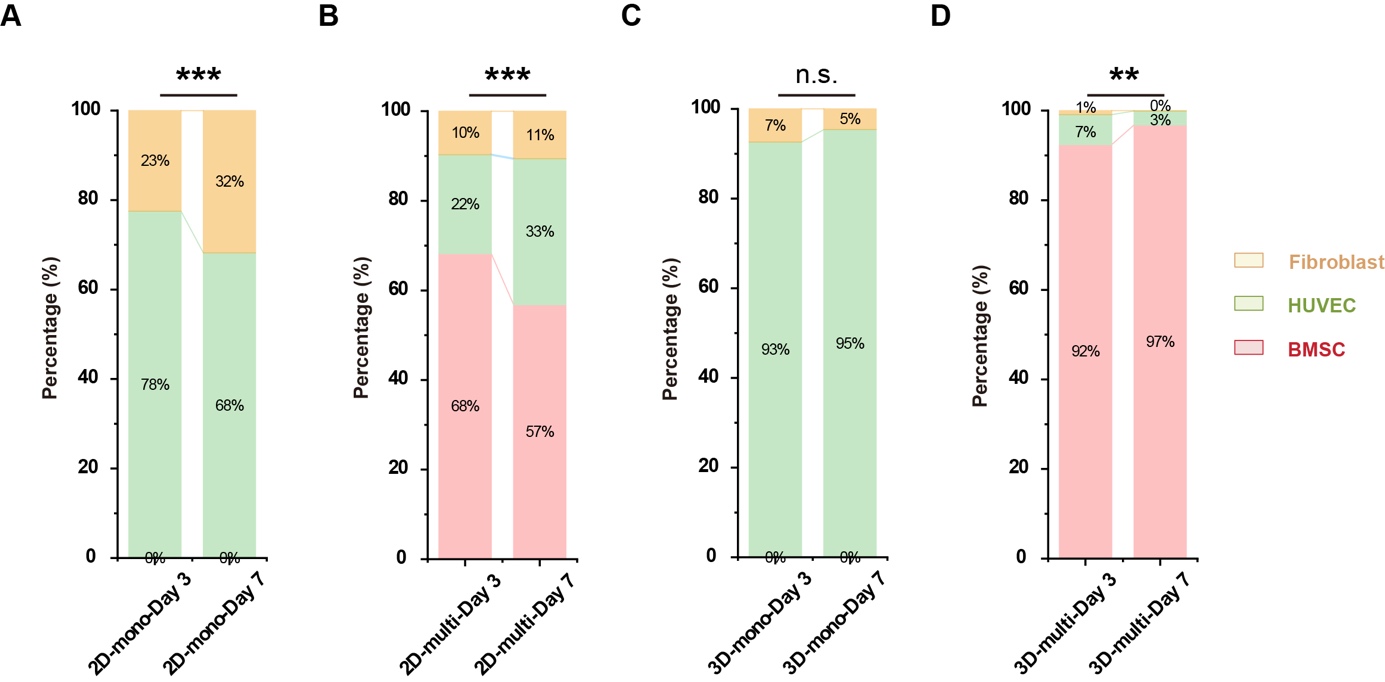


**Fig. S13.** A-D) Quantitative measurement of fibroblast infiltration test using a transwell system on days 3 and 7. All statistical data are represented as mean ± SEM. **P < 0.01, ***P < 0.001. Statistical analyses were performed using an unpaired two-tailed Student's t-test.


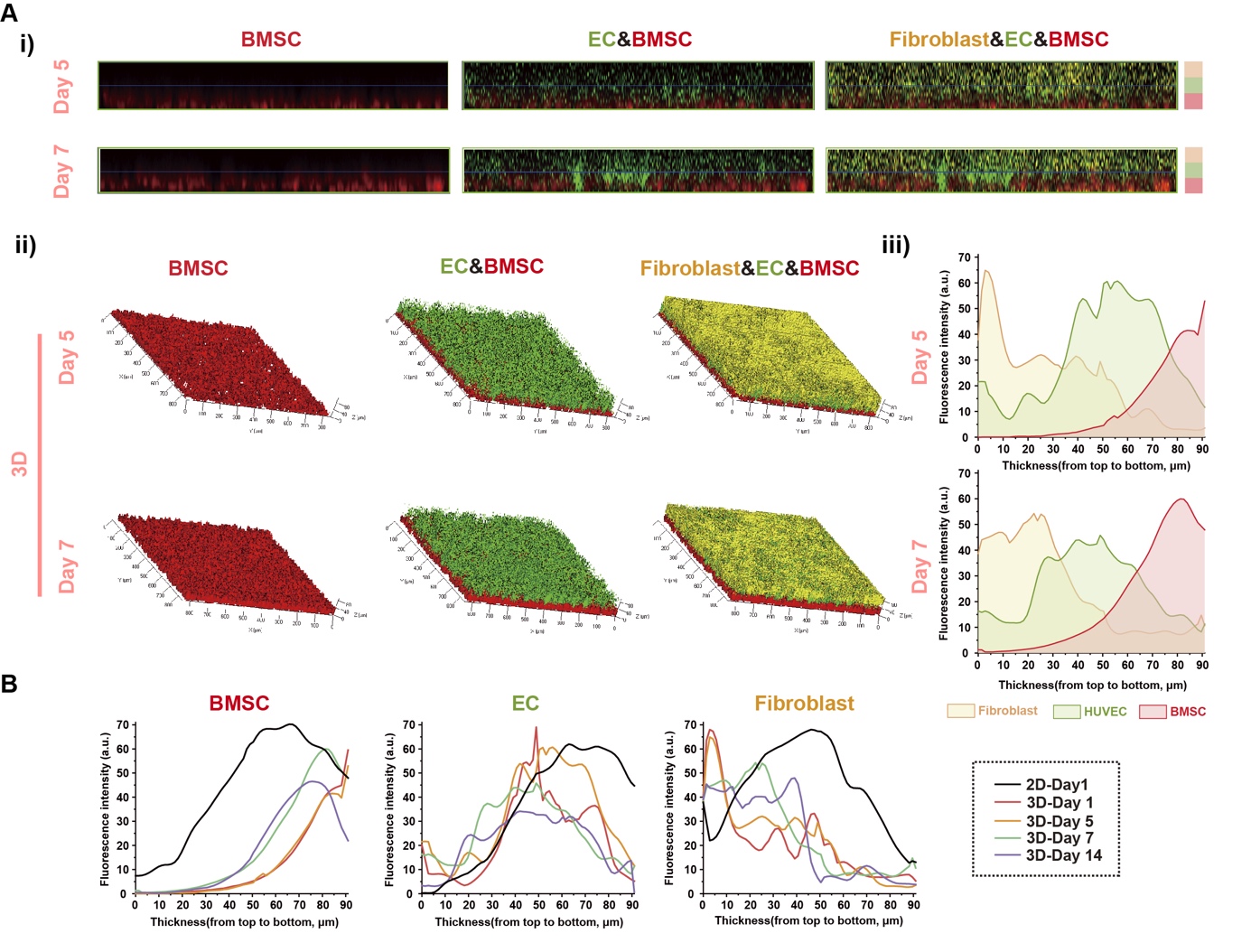


**Fig. S14.** Ai) 2D and Aii) 3D confocal images of ECs in 3D groups on days 5 and 7, and Aiii) the corresponding quantitative measurement. B) The quantitative measurement of BMSC, EC, and Fibroblast according to the confocal images.


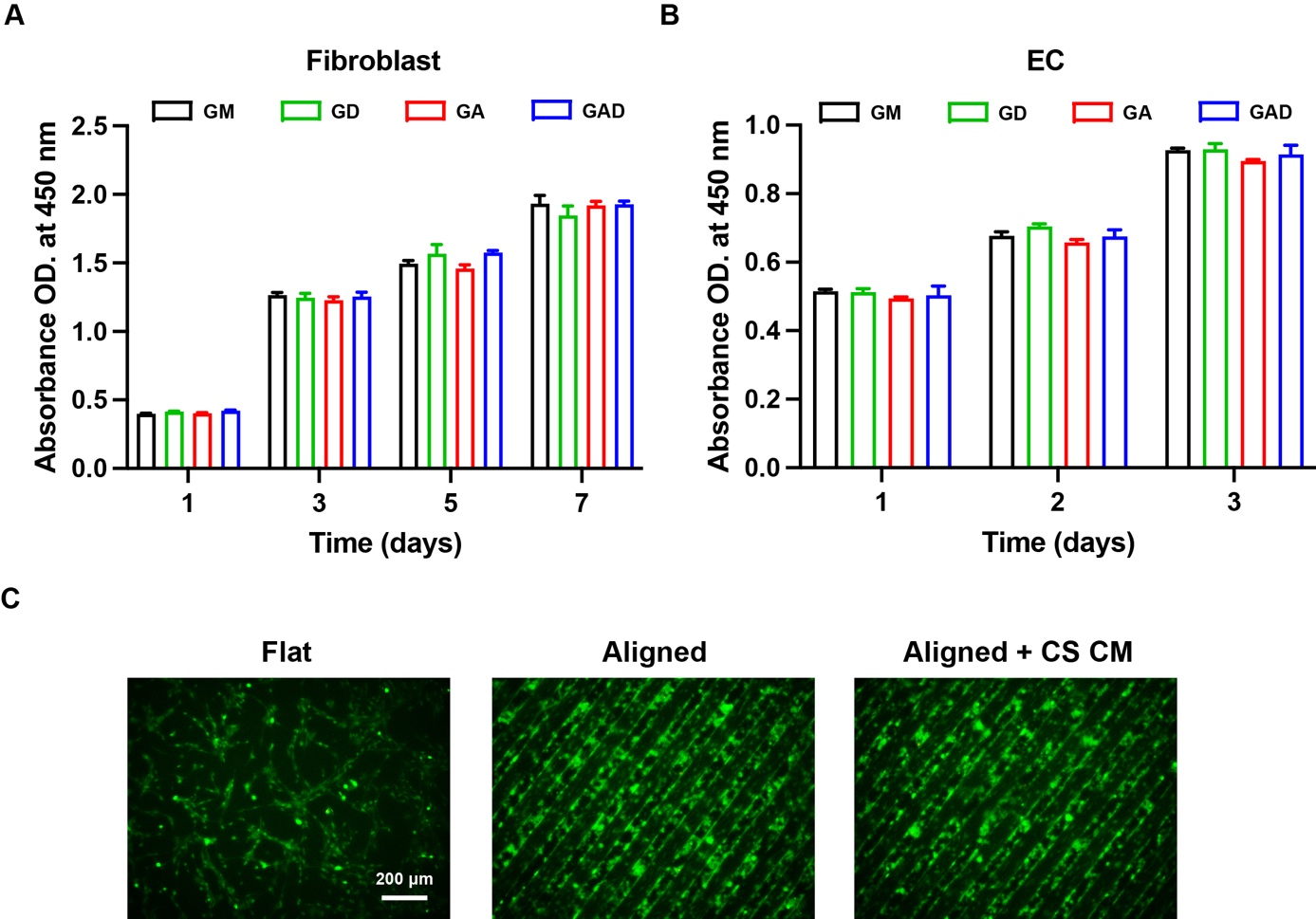


**Fig. S15.** Cytocompatibility of cells cultured with different groups of hydrogels. A) CCK8 analysis of fibroblasts seeded on various hydrogels for 1, 3, 5, and 7 days (n = 3). B) CCK8 analysis of ECs seeded on various hydrogels for 1, 2, and 3 days (n = 3). GM: GelMA, GD: GelMA with DOPA, GA: GelMA with sodium alginate, GAD: GelMA with sodium alginate and DOPA. C）Live/dead staining of fibroblasts seeded on various hydrogels on day 3 (Green: live cells; red: dead cells). All statistical data are represented as mean ± SEM.

Supplementary Tables:

**Table S1:** Primer sequences used for PCR analyses in this study.

| **Gene** | **Species** | **Primers** |
| --- | --- | --- |
| GAPDH | Rattus norvegicus | Forward: 5’-AGTGCCAGCCTCGTCTCATA-3’ |
|  |  | Reverse: 5’-GATGGTGATGGGTTTCCCGT-3’ |
| ALP | Rattus norvegicus | Forward:5’-CACGGCGTCCATGAGCAGAAC-3’ |
|  |  | Reverse: 5’- CAGGCACAGTGGTCAAGGTTGG-3’ |
| OSX | Rattus norvegicus | Forward: 5’-GTCCTGGCAACACTCCTACC-3’ |
|  |  | Reverse: 5’-GGGCAAAGTCAGACGGGTAA-3’ |
| OCN | Rattus norvegicus | Forward: 5’-TCGAGTCTGTCCATTGTGGTG-3’ |
|  |  | Reverse: 5’-ACGTGTCAGCAACTCTGTCT-3’ |
| OPN | Rattus norvegicus | Forward: 5’-AAGCCAGCCAAGGACCAACTA-3’ |
|  |  | Reverse: 5’- CTGCCAAACTCAGCCACTTTC -3’ |
| RUNX2 | Rattus norvegicus | Forward: 5’-TGGCCGGGAATGATGAGAAC-3’ |
|  |  | Reverse: 5’-TGAAACTCTTGCCTCGTCCG-3’ |

**Table S2:** Antibodies used in this study.

| **Antibodies** | **Source** | **Dilution** |
| --- | --- | --- |
| HIF-1α | GB111339-100, Affinity Biosciences | WB：1:2000 |
| VEGF | GB11034B-100, Affinity Biosciences | WB：1:1000 |
| ALP | DF6225, Affinity Biosciences | WB：1:1000  IHC：1:100 |
| RUNX2 | bs-1134R, Bioss | WB：1：1000 |
| CD31 | 28083-1-AP, Proteintech | IF：1:400 |
| OSX | DF7731, Affinity Biosciences | IF：1:200  IHC：1:100 |
| EMCN | 67854-1-Ig, Proteintech | IF：1:400 |
